# Estimation and Identifiability of Model Parameters in Human Nociceptive Processing Using Yes-No Detection Responses to Electrocutaneous Stimulation

**DOI:** 10.3389/fpsyg.2016.01884

**Published:** 2016-12-05

**Authors:** Huan Yang, Hil G. E. Meijer, Jan R. Buitenweg, Stephan A. van Gils

**Affiliations:** ^1^Applied Analysis, MIRA Institute for Technical Medicine and Biomedical Technology, University of TwenteEnschede, Netherlands; ^2^Biomedical Signals and Systems, MIRA Institute for Technical Medicine and Biomedical Technology, University of TwenteEnschede, Netherlands

**Keywords:** nociceptive processing, quantitative sensory testing, parameter estimation, parameter identifiability, model-based experiments

## Abstract

Healthy or pathological states of nociceptive subsystems determine different stimulus-response relations measured from quantitative sensory testing. In turn, stimulus-response measurements may be used to assess these states. In a recently developed computational model, six model parameters characterize activation of nerve endings and spinal neurons. However, both model nonlinearity and limited information in yes-no detection responses to electrocutaneous stimuli challenge to estimate model parameters. Here, we address the question whether and how one can overcome these difficulties for reliable parameter estimation. First, we fit the computational model to experimental stimulus-response pairs by maximizing the likelihood. To evaluate the balance between model fit and complexity, i.e., the number of model parameters, we evaluate the Bayesian Information Criterion. We find that the computational model is better than a conventional logistic model regarding the balance. Second, our theoretical analysis suggests to vary the pulse width among applied stimuli as a necessary condition to prevent structural non-identifiability. In addition, the numerically implemented profile likelihood approach reveals structural and practical non-identifiability. Our model-based approach with integration of psychophysical measurements can be useful for a reliable assessment of states of the nociceptive system.

## 1. Introduction

The human nociceptive system provides the neurophysiological basis of pain sensation. Following injury or disease, changes in peripheral, and central subsystems could lead to abnormal nociceptive function, e.g., hyperalgesia (Sandkühler, 2009). Long-term alterations in different subsystems can cause persistent pain, reducing quality of life (Voscopoulos and Lema, 2010). Efficient treatments could benefit from improved diagnosis of states of relevant nociceptive subsystems (Woolf and Max, 2001; Wilder-Smith, 2002; Arendt-Nielsen and Curatolo, 2013). Such a differential diagnosis might be achieved by reliably estimating physiologically meaningful parameters of the nociceptive system.

Several experimental paradigms have been proposed to provide information about states of nociceptive subsystems. For example, high throughput technologies can provide rich data about protein expression from pain-related biochemical networks in dissected tissue of animals (Niederberger and Geisslinger, 2008). For clinical practice on human patients, more efforts are still needed to transfer insights from those animal studies. In contrast, psychophysical approaches, e.g., quantitative sensory testing (QST) are non-invasive and can be conducted on human subjects within limited time (Cruz-Almeida and Fillingim, 2014). This makes QST useful for diagnosis of the nociceptive system and its malfunctioning in clinical practice (Walk et al., 2009). However, to dissect contributions of different nociceptive mechanisms, both a well-designed stimulus modality and physiology-based interpretation of limited QST measurements are required.

In general, the human nociceptive pathway first involves peripheral Aδ and C nociceptors. When these nociceptors are activated, their fibers convey the nociceptive information to neurons in the dorsal horn, where resultant activation is transmitted to supraspinal sites. To specifically assess Aδ-fiber-mediated nociceptive function, one can apply low-intensity electrocutaneous stimulation with an intra-epidermal needle electrode (Inui et al., 2002; Doll et al., 2014). The use of low amplitudes is compatible with a detection task (Doll et al., 2014; Yang et al., 2015), where delivered stimuli are pulse trains characterized by four controllable stimulus properties: the amplitude (*A*), the number of pulses (*NoP*), the inter-pulse interval (*IPI*), and the pulse width (*PW*). This newly developed experimental paradigm enables to measure various stimulus-response relations by applying stimuli with various values of stimulus properties, collecting a set of binary responses to electrocutaneous stimuli. This imposes a requirement to choose their values by considering time scales of peripheral and central nociceptive subsystems. First, a single-pulse stimulus with a relatively short *PW* (<1 ms) can recruit Aδ nociceptors without repetitive recruitment. Varying the *PW* adjusts the strength of overall peripheral activation, i.e., the number of recruited fibers. Changing the stimulus amplitude can also adjust this strength. In contrast to single-pulse stimuli, a pulse-train stimulus introduces temporal summation of the post-synaptic neuronal activity (van der Heide et al., 2009). Furthermore, Gescheider et al. (1999) reported a decreased detection threshold of vibro-tactile stimuli when decreasing *IPI*. The same authors explained that due to superposition of neural responses. To diagnose sensory function, conventional studies focus on psychophysical characteristics, e.g., detecting hyperalgesia by observing decreased thresholds (Walk et al., 2009). These thresholds are often determined by using a two-coefficient logistic psychometric function, which describes the detection probability with respect to the amplitude with fixed temporal properties (Treutwein, 1995; Doll et al., 2014). As a generalized linear model, in general, logistic regression can uniquely determine all regression coefficients and the resultant detection threshold. However, as this model does not straightforwardly account for sensory mechanisms, further efforts are required to interpret these intermediate estimates. In view of a variety of different proposed psychometric curves (Treutwein, 1995), the validity of the logistic curve is questionable, hampering further physiological interpretability of threshold estimates. In addition, more coefficients will be introduced, as the number of different combinations of temporal stimulus properties increases, yielding a potential overfit to a limited number of stimulus-response pairs.

In contrast to this conventional approach, we recently proposed two computational models to represent essential mechanisms in both peripheral and central nociceptive subsystems (Yang et al., 2015). Their states are characterized by six model parameters. The physiologically meaningful interpretation of these parameters offers potential for a mechanism-based diagnosis of the states of nociceptive subsystems. Our previous studies demonstrated qualitative agreements between model-based thresholds and experimental thresholds (Yang et al., 2015, 2016). Of the two models, the probabilistic hazard model (HM) is computationally more convenient for parameter estimation. Furthermore, the number of parameters in the HM is constant regardless of the stimulus set, i.e., all combinations of stimulus properties. Fitting a dataset measured from a detection task with four combinations of temporal parameters, the HM is a simpler model than the logistic regression model with eight regression coefficients. Also, the model-based psychometric function could substantially differ from a symmetric logistic function. However, it has not been studied whether the logistic regression model and the HM have different fits to experimental datasets.

Given reasonable replication of data by one model, reliable estimation is desired for the purpose of diagnosis and further usage of the parameter estimates. The uncertainty of estimates could be substantial, hampering assessment of the states of nociceptive subsystems. To address this uncertainty, parameter identifiability analysis is essential. Parameter non-identifiability is classified as structural and practical non-identifiability, which have different causes. The former manifests itself as non-unique estimates of model parameters. Equivalently speaking, changes in some non-identifiable parameters can be compensated by changes in other parameters, yielding equally optimal fits to data regardless of measurement accuracy. To analyze structural identifiability, most approaches are based on differential algebra (Ljung and Glad, 1994; Bellu et al., 2007). Based on Lie algebra, a recent study (Merkt et al., 2015) argued that structural non-identifiablity results from symmetries in differential equations with time-varying measurements. In case of a structurally non-identifiable model, one can resolve this by reducing or rebuilding the model. Alternatively, one can enrich measurements to eliminate structural non-identifiability. For that, persistence of excitation of subsystems is required for input signals (Miao et al., 2011). In contrast to structural non-identifiability, practical non-identifiability arises from limited information in data contaminated with noise. Hence, approaches based on differential-algebra are not applicable to assess practical identifiability. In contrast, the profile likelihood (PL) approach can be applied to real data to reveal both structural and practical non-identifiability (Raue et al., 2009, 2014).

For the nociceptive detection task, first, no study has evaluated and compared the abilities of both the logistic regression model and the HM to replicate measured data. Model comparison should consider the balance between model fit and complexity, i.e., different numbers of parameters for each model. For this, the Bayesian Information Criterion (BIC) is applicable (Kass and Raftery, 1995; Kingdom and Prins, 2010). Second, parameter identifiability has not been addressed for the nonlinear HM using binary detection responses to electrocutaneous stimuli from human subjects. To prevent structural non-identifiability, one should formulate prerequisites for the stimulus set. In addition, a limited number of measured data points could cause practical non-identifiability for model parameters. The pragmatic PL approach is expected to reveal parameter identifiability for the HM using binary detection responses to electrocutaneous stimuli.

To address these challenges, we start with a brief description of the experimental data, the logistic regression model and the HM. Next, we optimize their model fits to the data by maximizing the likelihood. We check the goodness of fit of the HM for multiple datasets. Based on the BIC, we assess the balance between model fit and complexity for the HM, and compare this with the logistic regression model. After that, we employ the profile likelihood approach to assess parameter identifiability, where some necessary conditions on stimulus sets for structurally identifiable models are formulated based on theoretical analysis.

## 2. Experiments and models

We consider a psychophysical experiment with datasets measured from healthy human subjects, focusing on Aδ-fiber-mediated nociceptive subsystems. We outline how we analyze these datasets. Next, we describe a conventional logistic regression model to fit stimulus-response pairs. After that, we briefly introduce the computational model representing both peripheral and central nociceptive subsystems.

### 2.1. Nociceptive detection task and datasets with stimulus-response pairs

A single detection experiment lasts for 10 min, providing around 200 stimulus-response pairs {*S, R*}, where *S* is the electrocutaneous stimulus and *R* is a binary response: detected (*R* = 1) or not-detected (*R* = 0). We refer to this collected set of binary responses to electrocutaneous stimuli as an elementary dataset. Stimulus *S* is delivered by an intra-epidermal needle electrode (Doll et al., 2014). For each applied stimulus, together with the current amplitude (*A*), three temporal stimulus properties: the number of pulses (*NoP*), the interpulse interval (*IPI*) and the pulse width (*PW*), determine the pulse train. In each 10 min experiment, four different combinations of temporal properties were used. For the same combination (*NoP*, *IPI*, *PW*), amplitudes were selected according to an adaptive probing procedure (Doll et al., 2014, 2016). For each combination, the number of stimuli was equal. Depending on whether each temporal property varied among four combinations, there are two designs of stimulus sets: with or without a variation of the *PW* denoted by *TS*_1_ and *TS*_2_, respectively, see Table 1.

**Table 1:** **Two designs with four combinations of temporal stimulus properties for the electrocutaneous pulse-train stimulus**.

**Design**	**Index**	**A**	**B**	**C**	**D**


*TS*_1_	*NoP*[#]	1	1	2	2
	*IPI*[ms]	–	–	10	50
	*PW*[ms]	0.42	0.84	0.42	0.42
*TS*_2_	*NoP*[#]	1	2	2	2
	*IPI*[ms]	–	10	50	100
	*PW*[ms]	0.42	0.42	0.42	0.42

*If NoP = 1, then the IPI is undefined*.

Using these two designs, two different groups of 15 healthy subjects were recruited, respectively (Doll et al., 2016). For each subject, the above-described 10 min detection experiment was conducted on 2 consecutive days, which we refer to as Day 1 and 2. We note that these datasets have been published in the previous studies to investigate effect of temporal parameters to either detection threshold (Yang et al., 2015) or detection probability (Doll et al., 2016) on a group level. As motivated above, our work focuses on mechanism-based assessment of the system parameters instead of merely psychophysical terms, e.g., detection thresholds.

Here, we briefly describe how we will use these datasets in the following sections. For the design with *TS*_1_, we consider all available 30 datasets, i.e., 2 for each of 15 subjects, to address the ability how well the logistic regression model and the hazard model replicate data. To address estimation and identifiability of parameters of the HM, we start with 15 elementary datasets measured on Day 1. For the nonlinear HM, estimation and identifiability of parameters could be hampered by the limited amount of information from a single 10 min experiment. For such cases, we propose criteria to combine datasets from Day 2 with their counterparts of Day 1 for the same subjects for further estimation. For the design with *TS*_2_, we only use one representative dataset (measured from subject D9001 on Day 1) to demonstrate structural non-identifiability together with a theoretical analysis in the section of identifiability analysis. In total, we consider 31 elementary datasets.

### 2.2. The logistic regression model

By convention, psychophysical studies often employ logistic regression to study the stimulus-response relation (Treutwein, 1995). With fixed stimulus properties *NoP*, *IPI*, and *PW*, the detection probability Pr(*R* = 1|*S*) is proposed to be a logistic function of the applied amplitude *A* (Doll et al., 2014). The pair of regression coefficients β_0_ and β_1_ characterizes the logit transformation as,

(1) $$\begin{array}{l} \text{logit}\left(\text{Pr}\left(R=1|S\right)\right)\;:=\;\log\left(\frac{\text{Pr}\left(R=1|S\right)}{1-\text{Pr}\left(R=1|S\right)}\right)=\beta_{0}+\beta_{1}A. \end{array}$$

Considering four combinations of temporal properties in *TS*_1_, the logistic regression model contains eight regression coefficients $\beta :=\cup_{j}\left\{\beta_{0}^{j},\beta_{1}^{j}\right\}$, with *j* = 1, 2, 3, 4 corresponding to four combinations A–D of *TS*_1_ see Table 1. Each pair of $\beta_{0}^{j}$ and $\beta_{1}^{j}$ characterizes the logit of the detection probability as,

(2) $$\begin{array}{l} \text{logit}\left(\text{Pr}\left(R=1|S_{ji}\right)\right)=\beta_{0}^{j}+\beta_{1}^{j}A_{ji}. \end{array}$$

where *A*_*ji*_ is the applied amplitude of the stimulus *S*_*ji*_ with the *j*th combination of temporal stimulus properties. Given a set of stimulus-response pairs, the coefficients can be determined using the glmfit routine in MATLAB.

### 2.3. The hazard model

We denote the model-based psychometric function, i.e., the conditional probability to detect stimulus *S*, as **Ψ**(*S*): = Pr(*R* = 1|*S*). A single trial may be simulated by drawing a random number ξ from a standard uniform distribution. The response is *R* = 1 when ξ < Ψ(*S*), indicating that the stimulus is detected, and *R* = 0 otherwise.

Here, we briefly describe the model, for more details see (Yang et al., 2015). Peripheral activation by the electrical stimulus is described by the threshold-linear function

(3) $$\begin{array}{l} {\left[f_{A}-\alpha_{1}\right]}_{+}\;:=\;\pi \left(f_{A}-\alpha_{1}\right)H\left(f_{A}-\alpha_{1}\right), \end{array}$$

where

(4) $$\begin{array}{l} f_{A}\;:=\;A\left(1-\exp\left(-\frac{PW}{\tau_{1}}\right)\right), \end{array}$$

and *H*(·) is a Heaviside step function; τ_1_ and α_1_ are the time constant and the activation threshold of afferent fibers, respectively. Next, through synaptic connections, an excitatory post-synaptic current $I_{p}^{*}\left(t\right)$ is induced

(5) $$\begin{array}{l} I_{p}^{*}\left(t\right)=\frac{{\left[f_{A}-\alpha_{1}\right]}_{+}}{\tau_{s}}\overset{NoP-1}{\underset{k=0}{\sum }}\exp\left(-\frac{t-kIPI}{\tau_{s}}\right)H\left(t\right), \end{array}$$

with time constant τ_*s*_ = 1.5 ms (Gabbiani et al., 1994). This drives the post-synaptic potential *x*(*t*) of a secondary dorsal horn neuron, which we model as a leaky integrator

(6) $$\begin{array}{l} \tau_{2}\dot{x}=-x+I_{p}^{*}\left(t\right),\;x\left(0\right)=0. \end{array}$$

The value of the time constant τ_2_ is roughly several tens of milliseconds (Prescott and Koninck, 2002; Weng et al., 2006). This noise-free post-synaptic potential is converted into an instantaneous firing rate through a non-homogeneous Poisson process (Plesser and Gerstner, 2000)

(7) $$\begin{array}{l} \lambda \left(t\right)=\lambda_{L}{\left(1+\exp\left(\frac{\alpha_{L}-x\left(t\right)}{\sigma_{L}}\right)\right)}^{-1}. \end{array}$$

Here, the lumped parameters α_*L*_, σ_*L*_, and λ_*L*_ represent the threshold, the slope parameter and the maximal firing rate, respectively. The expected value of the number of spikes during a trial interval of duration *T* is,

(8) $$\begin{array}{l} \lambda_{T}=\int_{0}^{T}\lambda \left(t\right)dt. \end{array}$$

The binary response *R* equals one given sufficient activity in the central nociceptive subsystem. We assume that sufficient activation implies at least one secondary neuron generated an action potential during the trial interval *T*. So the model-based psychometric function evaluated at the parameters **θ** is given by

(9) $$\begin{array}{l} \Psi_{\theta }=1-\exp\left(-\lambda_{T}\right). \end{array}$$

As Equation(6) is linear, we obtain an analytical, but complex, formula for Ψ_θ_.

**Figure 1:**
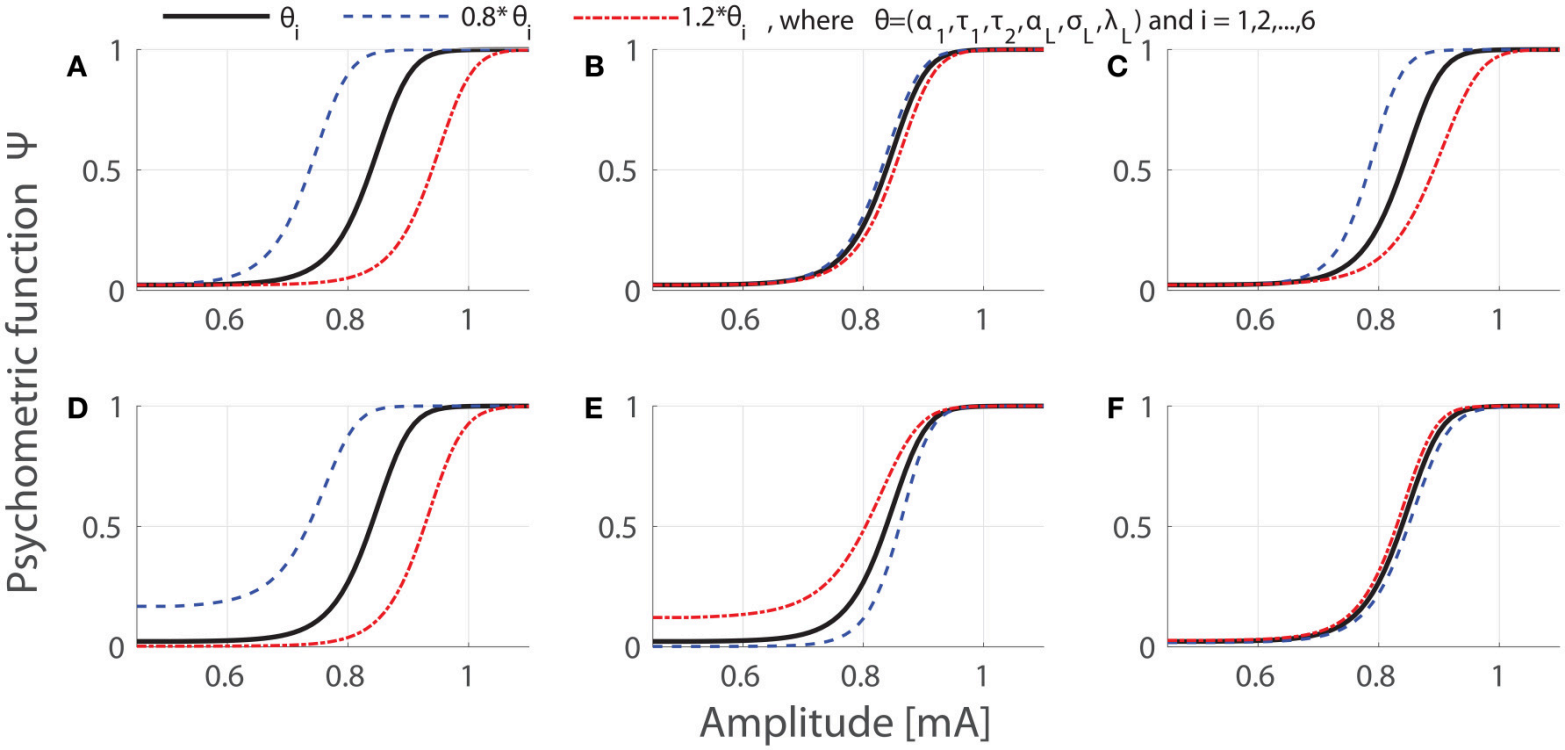
**Effects of parameter variations on the psychometric curves**. We set temporal properties of stimulation as *NoP* = 1 and *PW* = 0.42 ms. In each panel, psychometric curves evaluated at reference values, decreased or increased values of single parameters are shown in black, blue or red, respectively. The panel indices **(A–F)** correspond to indices 1–6 for single parameters, respectively.

Our model contains six lumped parameters **θ** = (α_1_, τ_1_, τ_2_, α_*L*_, σ_*L*_, λ_*L*_), which depend on more than 10 physical quantities, characterizing peripheral, and central nociceptive components. Regarding meaning of lumped parameters, α_1_ and τ_1_ merely quantify peripheral characteristics, and τ_2_ and λ_*L*_ merely describe the central properties. But α_*L*_ and σ_*L*_ are compound characteristics of both subsystems. Figure 1 illustrates the dependence of the psychometric curves on parameters. We vary values of single lumped parameters to either 0.8 or 1.2 fold of their reference values, which was set as **θ** = (α_1_ = 0.1, τ_1_ = 0.1, τ_2_ = 50, α_*L*_ = 0.022, σ_*L*_ = 0.0021, λ_*L*_ = 0.4020) as used in Yang et al. (2015). We remark that the psychometric functions are monotone with respect to three parameters: α_1_, τ_1_, and α_*L*_. In addition, as known from model development in Yang et al. (2015), the obvious functional dependence makes most of physical quantities non-identifiable. Hence in our work, we focus on estimation and identifiability of the lumped parameters rather than physical quantities.

## 3. Model fitting and evaluation

Here, we formulate the likelihood function based on binary responses. We achieve an optimal fit of the HM by maximizing the likelihood function and check the goodness of fit to data. To evaluate the balance between model fit and complexity, we compute and compare the Bayesian Information Criterion of the HM and the logistic regression model.

### 3.1. Optimal model fitting of the HM by maximizing the likelihood function

To quantify the goodness of model fit to data, the likelihood is given by

(10) $$\begin{array}{l} L\left(\theta \right)=\text{Pr}\left(Data|\theta \right), \end{array}$$

where the *Data* contain a sequence of stimuli and binary responses. The inter-stimulus interval varied from 2 to 5 s which is much larger than the time constants in the nociceptive system (Mogyoros et al., 1996; Prescott and Koninck, 2002; Weng et al., 2006). Hence, we assume the trials were independent. Grouping all stimuli with the same stimulus properties, we compute the binomial coefficient from the corresponding responses, and its confidence interval (Clopper and Pearson, 1934).

**Figure 2:**
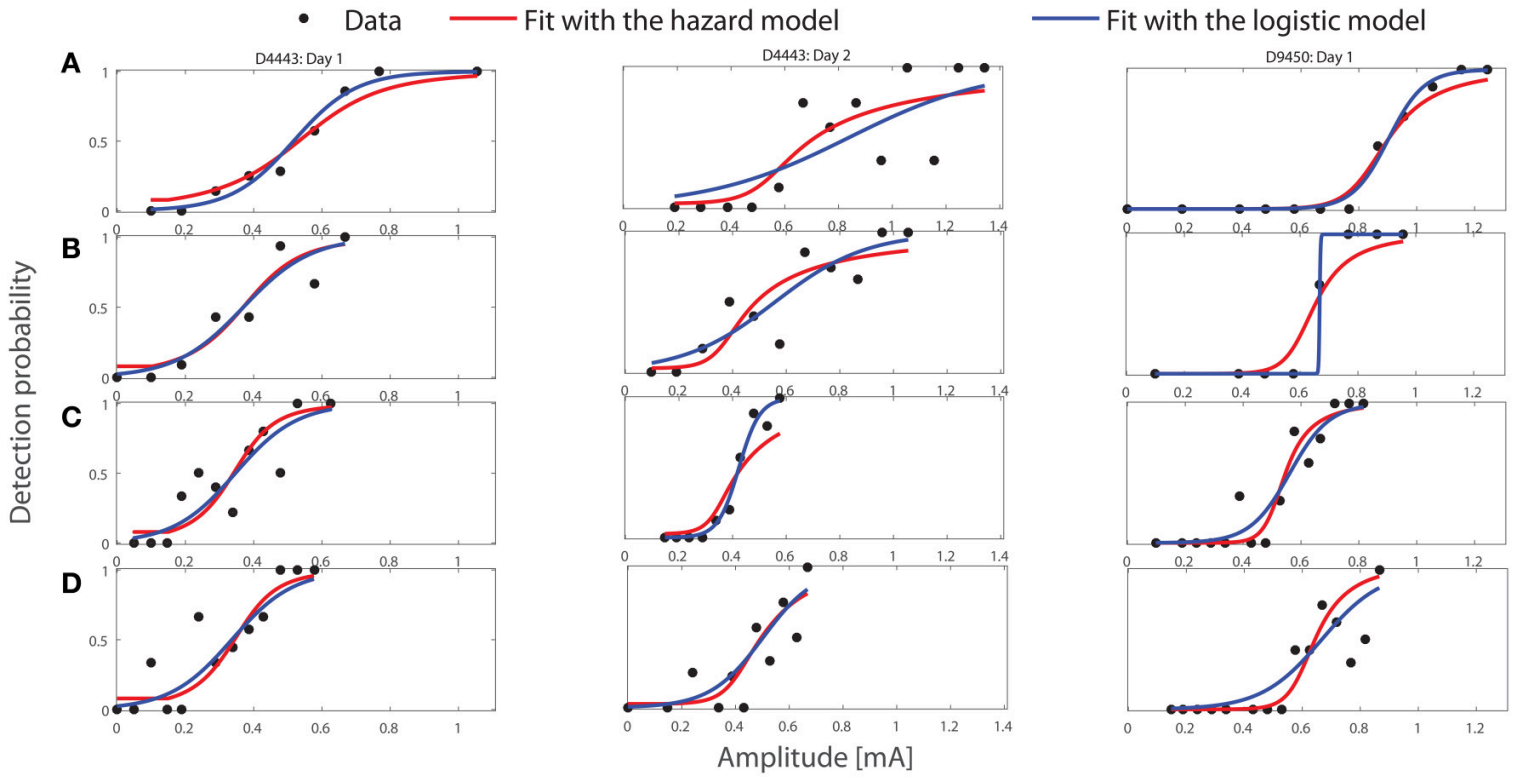
**Fitting performance of the HM and the logistic model with the optimal values of parameters to three representative sets of experimental stimulus-response pairs using ***TS***_**1**_, see Table 1**. The titles of three columns indicate the subject IDs and the measurement day. Panels **(A–D)** correspond to the combinations A–D of design *TS*_1_ in Table 1.

As the same stimulus amplitudes could be applied multiple times, we represent the detection probability as a function of the amplitude, see the black dots in Figure 2. The independence among trials simplifies the likelihood (Equation 10) as

(11) $$\begin{array}{l} L\left(\theta \right)=\overset{n_{D}}{\underset{k=1}{\prod }}\Psi_{k}^{R_{k}}{\left(1-\Psi_{k}\right)}^{1-R_{k}}, \end{array}$$

where Ψ_*k*_ is the model-based psychometric function value for the applied stimulus *S*_*k*_ evaluated at **θ**, and *k* = 1, 2, …, *n*_*D*_ with *n*_*D*_ the total number of stimuli. By maximizing the likelihood function, one obtains the optimal values of parameters

(12) $$\begin{array}{l} \hat{\theta }={\text{argmax}}_{\theta \in \Theta }L\left(\theta \right)={\text{argmin}}_{\theta \in \Theta }-\log L\left(\theta \right), \end{array}$$

where **Θ** is the parameter space. We restrict **Θ** to be a hypercubic domain with the lower and upper boundaries of model parameters given in Table 2. Our choice of the boundaries for τ_1_, τ_2_, and λ_*L*_ is based on experimental studies (Mogyoros et al., 1996; Prescott and Koninck, 2002; Weng et al., 2006). For the other three, their lower boundaries approach zero. We set the upper boundary of α_1_ to one. We motivate this choice as when α_1_ > 0, the threshold-linear function (Equation 3) hardly produces effective activation for the applied amplitudes contrary to the observed dependency of detection probability on amplitudes, see Figure 2. The upper boundaries of α_*L*_ and σ_*L*_ are set to relatively high values.

**Table 2:** **Lower and upper boundaries of the feasible parameter space**.

**Model parameter**	**α_1_ [mA]**	**τ_1_ [ms]**	**τ_2_ [ms]**	**α_*L*_ [A/s]**	**σ_*L*_ [A/s]**	**λ_*L*_ [kHz]**
Lower boundary	10^−6^	10^−2^	2	10^−5^	10^−8^	10^−3^
Upper boundary	1	3	10^3^	1	0.1	10^2^

Given the nonlinear nature of the HM, multiple local optima of parameters could exist in the likelihood landscape. To find the global optimum, we employ a Monte Carlo method with multiple starting values. We generate a set of *N*_*s*_ = 100 starting values {**θ**_*s*_} in **Θ** using Latin hypercube sampling (McKay et al., 2000). For each starting value, we use Trust-Region-Reflective-Newton method to obtain the local minimum **θ**_*f*_ (Coleman and Li, 1996). After applying this local optimization for all starting values, we take the estimate $\hat{\theta }=\theta_{f}$ with the minimal −log *L*(**θ**_*f*_) according to Equation (12). This numerical optimization is implemented in MATLAB 2014b with the lsqnonlin routine. In our study, the lsqnonlin routine is applicable for this optimization purpose, because the negative log-likelihood can be formulated as a sum of square of components, where each component is a psychometric function value. Each component can be rewritten as square of the square root of the psychometric function value (or one minus this value). In this way, it can be considered as a nonlinear least squares problem. It is also possible to implement the optimization with the fmincon routine. We prepare matlab scripts to compare the two routines, see the folder Comparison_fmincon_lsqnonlin in Supplementary Material 2. Here, we give several remarks regarding optimal model fitting and parameter estimation.

First, the local optimization should be efficient, so that the ranked likelihood after optimization shows a step-like pattern (Raue et al., 2013). We observe such patterns in our estimation results based on experimental data, see Supplementary Figure 3.

Second, this multiple-starting-value optimization scheme with *N*_*s*_ = 100 does not necessarily yield the optimal fit, i.e., the lowest −log(*L*). To check whether *N*_*s*_ = 100 is sufficiently large, one can sample more starting values, and re-optimize with those samples. If we do not observe any substantially more optimal fit, we consider *N*_*s*_ = 100 sufficient to obtain the optimal fit. In Section 4, we employ this strategy as a validation procedure by implementing a profile-likelihood approach with more samples within **Θ**.

Third, we clarify two aspects: how well a model replicates observations and how reliable parameter estimates are, although the optimal model fit and parameter estimates are always achieved simultaneously from Equation (12). On one hand, plausible models are expected to give a good fit to observations with a relatively small $-\log\left(\hat{L}\right)$. One can perform a likelihood-ratio-based test to evaluate goodness of fit to measurements, see e.g., García-Pérez and Alcalá-Quintana (2015a,b). The idea is to compare the purposed HM model and the saturated model (which contains parameters just being detection probabilities based on binomial fit at each amplitude) by the ratio of likelihood denoted by *G*^2^. The *p* computed from a χ^2^ distribution will inform whether the HM can fit the data. On the other hand, good replication of data does not imply sufficient identifiability of parameters. For nonlinear models with relatively small $-\log\left(\hat{L}\right)$, the reliability of parameter estimates could be questionable due to either limited amount of data or an insufficiently rich stimulus set (Raue et al., 2009). We address these two aspects separately. We apply the above-mentioned model fitting and parameter estimation to all 31 elementary datasets. With the obtained optimal fitting for data with *TS*_1_, we address model plausibility in the following subsection. Regarding reliable estimates, the first prerequisite is to obtain estimates **θ** not lying on the boundary of **Θ**. There are two influential factors: (i) the model structure and (ii) to what extent the observations from a single elementary experiment on Day 1 represent characteristics of nociceptive processing of the subject. In case estimates end up on the boundary of **Θ**, the model should be refined, which is beyond the scope of this work. Here, for the stimulus set with *TS*_1_, we try to improve the second factor by combining/adding measurements from the same subject on Day 2, if the two elementary datasets are qualitatively similar. We visually inspect the similarity of the stimulus-response pairs on Day 2 to those on Day 1. For this, we propose two exclusion criteria: either the applied amplitudes were clearly shifted or one or more detection probabilities differed without overlapping confidence intervals. With one combined dataset, we estimate parameters again and check whether they become interior estimates. In case of interior estimates using *TS*_1_, we further quantify parameter identifiability in section 4.

### 3.2. The balance between model fit and complexity

During the development of a computational model, one desires to obtain good model fit to experimental observations. We perform a likelihood-ratio-based goodness test to check how good the HM can replicate data. In addition, to prevent overfitting with too many free parameters, one needs to reduce the complexity during model development. To assess the balance between model fit and complexity, we propose to use Bayesian Information Criterion (Kingdom and Prins, 2010) given by

(13) $$\begin{array}{l} BIC=-2\;\log\left(\hat{L}\right)+n_{P}\;\log\left(n_{D}\right), \end{array}$$

where $\hat{L}$ is the optimal likelihood evaluated with parameter estimates $\hat{\beta }$ and $\hat{\theta }$ for the logistic regression model and the HM, respectively. In addition, *n*_*D*_ is the number of observations and *n*_*P*_ is the number of model parameters. For the logistic regression model (Equation 2), *n*_*P*_ = 8; for the HM, *n*_*P*_ = 6. The first term of the BIC in Equation (13) provides a measure of goodness of fit of the model to stimulus-response pairs. The second term penalizes the BIC for the number of parameters. BIC is a relative measure as the optimal likelihood and *n*_*P*_ depend on a specific dataset. Hence, for the purpose of model selection given the same dataset, the smaller the value of the BIC is, the more plausible the model is. We compute 30 pairs of BIC-values for both models with 30 elementary datasets with *TS*_1_. We check whether HM has a better balance of model fit and complexity with the hypothesis: the probability that HM has a smaller BIC than the logistic regression model is >0.5. For that, we perform a one-tail binomial test.

### 3.3. Results

First, we visualize the fit of the HM and the logistic model to three representative sets of experimental data using *TS*_1_. We present stimulus-response measurements in Figure 2. The black dots represent the experimental detection probabilities. The red curves show the predictions of detection probabilities based on $\hat{\theta }$ in the HM. The blue curves are the predictions based on the regressed coefficients $\hat{\beta }$ in the logistic regression model (Equation 2). Row labels A–D correspond to combinations A–D of *TS*_1_, respectively. In the Supplementary Material, we visualize the fitting performance of both models for all 30 datasets using *TS*_1_. According to computed statistics about the goodness of fit about the HM, there are only 2 of 30 cases with rejected fit, see Tables S1, S2 in the Supplementary Material.

**Figure 3:**
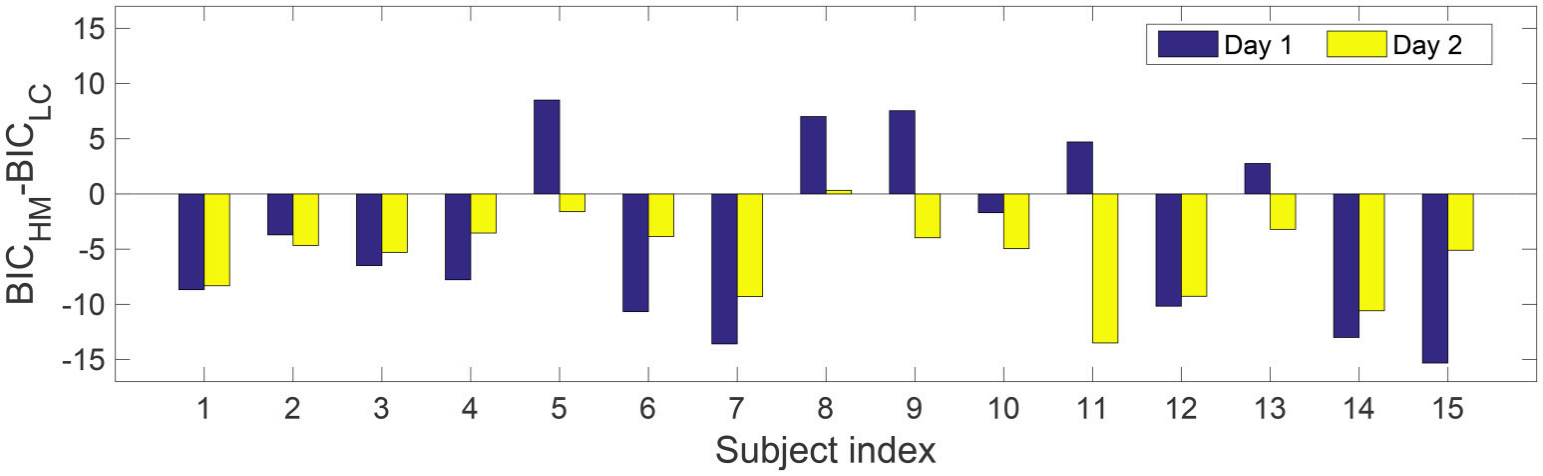
**Difference of Bayesian Information Criterion between the HM the logistic model with the measured dataset on the two study days**. For each single dataset, a negative difference suggests the HM is preferred, while the logistic model is preferred when the difference is positive. Note that each dataset contains about 200 data points without combining datasets from the same subject.

Second, Figure 3 shows difference in BIC-values between the HM and the logistic model for 30 datasets from 2-day experiments on 15 subjects. In case of a negative difference, the HM is preferred, while a positive difference favors the logistic model. There are 24 cases where the HM has a smaller value of BIC compared to the logistic regression model. The binomial test yields *p* < 0.001, suggesting that the HM is better than the logistic regression model.

Third, for estimation results using data on Day 1, we find that 10 out of 15 estimates lie on the boundary of **Θ**. After checking the similarity of 10 corresponding pairs of datasets, there are five cases where we can combine the pairs of datasets. For two of these five combined datasets, we obtain interior estimates $\hat{\theta }$. For the other three subjects, even with these additional datasets, the estimates still lie on the boundary of **Θ**. Hence, for the design with *TS*_1_, five elementary datasets on Day 1 and two combined datasets yield interior estimates, for which we will address parameter identifiability.

## 4. Identifiability analysis

Even though we obtained reasonable fit of the data to the HM, it is not guaranteed that the estimates of parameters are uniquely determined, i.e., identifiable. Here, we start with a brief description of the profile likelihood approach with emphasis on how it can reveal various types of (non-)identifiability. Next, we perform identifiability analysis for the hazard model in two aspects. First, a condition on combinations of temporal stimulus properties necessary for structural identifiability follows from a theoretical analysis. Equivalently speaking, we derive a sufficient condition for a structurally non-identifiable HM. In addition, we provide an analytically tractable quantification of the set identifiability of non-identifiable parameters, i.e., the range over which the structural non-identifiability exists. These findings are illustrated by results from the PL approach with one experimental dataset using *TS*_2_. Second, to address practical (non-)identifiability, we present the PL results for seven datasets using *TS*_1_. When practical non-identifiability occurs, we perform a model-based study to efficiently choose stimulus properties for improved parameter identifiability.

### 4.1. Profile likelihood approach

The profile likelihood is a univariate function with respect to each single parameter **θ**_*i*_ with *i* = 1, 2, …, *n*_*P*_,

(14) $$\begin{array}{l} PL\left(\theta_{i}\right)=\underset{\theta_{\left\{/i\right\}}\in \Theta_{\left\{/i\right\}}}{\max}L\left(\theta \right), \end{array}$$

where {/*i*}: = {1, 2, …, *i*−1, *i*+1, …, *n*_*P*_} and *n*_*P*_ is the number of parameters (Raue et al., 2009). Fixing one parameter at a specific value $\theta_{i}^{*}$, the value $PL\left(\theta_{i}^{*}\right)$ is determined as the maximal likelihood by tuning the remaining *n*_*P*_ − 1 parameters **θ**_{/*i*}_, i.e.,

(15) $$\begin{array}{l} {\hat{\theta }}_{\left\{/i\right\}}={\text{argmax}}_{\theta \in \Theta \text{ with }\theta_{i}=\theta_{i}^{*}}L\left(\theta \right). \end{array}$$

The details on the computation of the PL are given in Appendix A. One may notice that the formula of the PL (Equation 14) is similar to the formula to obtain the maximal likelihood (Equation 12). As more local optimizations are involved, the PL approach can be considered a validation procedure for the obtained estimates and optimal model fit.

Typically, the likelihood is nonzero around the maximum, because the observations are noisy and the uncertainty propagates into the parameter estimates. This could result in parameter uncertainty in the parameter space. For identifiability analysis, the PL approach is applicable to assess uncertainty of parameters of any (non)linear model, provided the likelihood is computable (Raue et al., 2009). Before we directly step to the nonlinear hazard model, we discuss how it works for a conventional linear regression model and then nonlinear models in general.

For linear regression, −2 log(*PL*) obeys a quadratic relation with a single regression coefficient. In the sequel, we define −2 log(*PL*) as *LPL*. For all coefficients, all parabolas have the same vertical value of vertices $-2\;\log\left(\hat{L}\right)$. The width of the parabola depends on the data quality. In general, increased data length or decreased noise strength will yield a narrower width. The narrower the parabola is, the less uncertain the estimate for the single coefficient is. In the other case, the parabola will become a horizontal line where the corresponding coefficient is non-identifiable. To quantify such uncertainty, one can determine confidence intervals of the coefficient due to the fact that *LPL* approximately follows the χ^2^-distribution with one degree of freedom (Uusipaikka, 2008). Given a desired significance level α, the confidence intervals of one particular coefficient are given by $\left\{\theta_{i}|-2\;\log\left(PL\left(\theta_{i}\right)\right)\leq -2\;\log\left(\hat{L}\right)+\chi_{\alpha }^{2}\right\}.$ When α = 0.95, the offset is $\chi_{\alpha }^{2}=3.84$. When α approaches zero, the lower and upper boundaries of the confidence interval coincide with the maximum likelihood estimate. For the extreme situation when $-2\;\log\left(\hat{L}\right)$ is a horizontal line over the entire range of that parameter, confidence intervals are always unbounded for any significance level. Note that this should be considered as structural non-identifiability for a linear regression model, as the *LPL* is either a horizontal line or a parabola opening upwards.

The shape of *LPL* helps to determine structural identifiability for a nonlinear model. In general, *LPL* could depend in a different way, i.e., not quadratically, on a single parameter. When a unique global minimum *LPL* for one parameter exists, this parameter is structurally identifiable. In contrast, structural non-identifiability manifests itself as multiple different values of **θ** with the same optimal fit, diminishing the value of the estimate for further use. Those minima may be isolated in the parameter space for a nonlinear model. However, in general situations, multiple global minima form some manifold in the parameter space. As a result, *LPL* plots of some parameters will be flat. In case that such a manifold extends to boundaries of some parameters, the flatness in corresponding *LPL* plots will span the entire range of parameters. In other cases, the flatness of the *LPL* plot spans a narrower interval for the corresponding parameter. For this, Schmidt (2015) used the concept of set identifiability to deal with parameters that are structurally identifiable only up to a specific interval. Here, we stress the similarity between conventional structural non-identifiability and set identifiability in terms of a flat *LPL*. So, our study considers set identifiability as one particular example of structural non-identifiability. Determining this range can help to understand the specific parameter identifiability. Given the model structure, one can study structural identifiability. Several studies derived sufficient or necessary conditions for structurally identifiable for models with specific structures (Gargash and Mital, 1980; Saccomani and Cobelli, 1993; Bazanella et al., 2012). For our hazard model, such sufficient and/or necessary conditions for structural identifiability have not been explored. On one hand, sufficient conditions can guarantee model identifiability, but such requirements could be too strict to be met in practice (Bellman and Åström, 1970; Chiş et al., 2011). On the other hand, finding necessary conditions prevents to measure input-output relations with insufficient excitation of subsystems, which is helpful before conducting expensive experiments, e.g., on human subjects.

Next, for structurally identifiable parameters, practical non-identifiability could arise from the limited amount of information from existing observations. Similar to the linear case, one can define the confidence interval as

(16) $$\begin{array}{l} CI_{\alpha }\left(\theta_{i}\right)=\left\{\theta_{i}\in \left(\theta_{i}^{l},\theta_{i}^{u}\right)|-2\;\log\left(PL\left(\theta_{i}\right)\right)\leq -2\;\log\left(\hat{L}\right)+\chi_{\alpha }^{2}\right\}, \end{array}$$

where $\theta_{i}^{l},\theta_{i}^{u}$ are the lower and upper boundaries of parameter θ_*i*_, respectively. Here, the probability α accounts for the true parameter value lies within the confidence interval based on profile likelihood (Kreutz et al., 2012). A χ^2^-distribution is used to approximate the profile-likelihood-based confidence interval, and the study (Kreutz et al., 2012) also checked PL-based intervals with empirically generated intervals from a Monte Carlo simulation. Here, we discuss a few cases for the six parameters in the hazard model. Moreover, we present all results in the Supplementary Material. In general, we showed that χ^2^-distribution can approximate the empirical one from simulations. When α → 0, the confidence interval becomes just the maximum-likelihood estimate. However, with a fixed α, when *LPL* was not restricted to the threshold $\chi_{\alpha }^{2}$, the parameter becomes practically non-identifiable. To address practical identifiability, we need to treat each dataset case by case, because the PL reflects the likelihood landscape.

### 4.2. Structural and practical non-identifiability in the HM

In the case of the nonlinear HM, structural and practical non-identifiability could occur. First, structural non-identifiability could arise from a qualitatively insufficient stimulus set, i.e., (*A*, *NoP*, *IPI*, *PW*) rather than observed stimulus-response pairs contaminated with noise. Such insufficiency results in non-unique parameter estimates. As one trivial example, when only amplitudes below the peripheral activation α_1_ are applied, this implies $A\left(1-\exp\left(-\frac{PW}{\tau_{1}}\right)\right)<\alpha_{1}$ for any value of *PW*. No matter how the combinations of temporal stimulus properties are chosen, one can expect that α_1_ is structurally non-identifiable due to over-parameterization in case of activation below threshold in Equation (3). Hence, a prerequisite for a structurally identifiable HM is to apply some amplitudes above α_1_. In addition to this trivial case, in Section 4.2.1, we derive analytically a necessary condition on the *PW* for structural identifiability. We also quantify the set identifiability for such a structurally non-identifiable case. To illustrate this structural non-identifiability, we show the numerically evaluated results using one experimental dataset in Section 4.2.2. Second, as practical identifiability depends on datasets, we perform the PL approach with seven cases with interior estimates using *TS*_1_, with numerical results in Section 4.2.2.

#### 4.2.1. Theoretical analysis for structural non-identifiability in the HM

In detection task with pulse-train stimuli, we use a relatively large *IPI* (≥ 10 ms) compared to the time constant for peripheral activation (Mogyoros et al., 1996). Then the *PW* is the only effective temporal property to control peripheral activation. In case that the *PW* is invariant for all stimuli, changes of parameters for central processing may be compensated by other parameter changes for peripheral activation. This hampers separation of their contributions to the overall nociceptive function, leading to non-identifiable parameters. Such a suspicion deserves a rigorous study for structural identifiability. There are two questions: whether a stimulus set with identical *PW* definitely introduces structurally non-identifiable parameters, i.e., is it necessary to use multiple values of *PW* for structural identifiability. If so, how to quantify the set identifiability for resultant structurally non-identifiable parameters? To answer the first, we perform a theoretical analysis with the hazard model. Our strategy is to search for a redundant quantity with respect to **θ**. The cascaded structure in the HM facilitates our search. By investigating peripheral activation (Equation 3) together with synaptic transmission (Equation 5), and processing and activation of secondary neurons (Equations 6, 7), one can find that redundancy of **θ** exists in the instantaneous firing rate for secondary neurons λ(*t*). For example, given two vectors of different parameter values **θ**^(1)^ and **θ**^(2)^ satisfying

(17) $$\begin{array}{l} \{\begin{array}{c} \frac{\alpha_{1}^{\left(1\right)}}{\sigma_{L}^{\left(1\right)}}\;=\;\frac{\alpha_{1}^{\left(2\right)}}{\sigma_{L}^{\left(2\right)}},\\ \frac{\alpha_{L}^{\left(1\right)}}{\sigma_{L}^{\left(1\right)}}\;=\;\frac{\alpha_{L}^{\left(2\right)}}{\sigma_{L}^{\left(2\right)}},\\ \frac{1-\text{exp }\left(-\frac{PW}{\tau_{1}^{\left(1\right)}}\right)}{\sigma_{L}^{\left(1\right)}}\;=\;\frac{1-\text{exp }\left(-\frac{PW}{\tau_{1}^{\left(2\right)}}\right)}{\sigma_{L}^{\left(2\right)}},\\ \tau_{2}^{\left(1\right)}=\tau_{2}^{\left(2\right)}\;,\;\lambda_{L}^{\left(1\right)}=\lambda_{L}^{\left(2\right)}, \end{array} \end{array}$$

the HM can produce the same λ(*t*) given any arbitrary values of *t*, *NoP*, and *IPI*. Given this, all quantities depending on λ(*t*), including the likelihood function (Equation 11), become redundant. Suppose that one obtains $\hat{\theta }$ from the numerical optimization in Section 3, according to Equation (17), a distinct parameter vector, i.e., a spurious solution, $\theta^{*}=\left(\alpha_{1}^{*},\tau_{1}^{*},\tau_{2}^{*},\alpha_{L}^{*},\sigma_{L}^{*},\lambda_{L}^{*}\right)$ with two restrictions:

(18) $$\begin{array}{l} (\text{i})\;\tau_{2}^{*}=\hat{\tau_{2}},\;\lambda_{L}^{*}=\hat{\lambda_{L}},\text{ and}\\ (ii)\frac{\alpha_{1}^{*}}{\hat{\alpha_{1}}}=\frac{\alpha_{L}^{*}}{\hat{\alpha_{L}}}=\frac{\sigma_{L}^{*}}{\hat{\sigma_{L}}}=\frac{1-\text{exp }\left(-\frac{PW}{\tau_{1}^{*}}\right)}{1-\text{exp }\left(-\frac{PW}{\hat{\tau_{1}}}\right)}, \end{array}$$

will have the same optimal fit. The restriction (i) in Equation (18) implies that the parameters τ_2_ and λ_*L*_ could be structurally identifiable. However, four parameters α_1_, α_*L*_, σ_*L*_, and τ_1_ in (ii) of Equation (18) are structurally non-identifiable. We conclude that it is necessary for structural identifiability to use multiple values of PW in the experimental stimulus parameters.

With detected structural non-identifiability, we investigate whether and how the restriction (ii) can affect the set identifiability. By denoting the ratio between the spurious values of the estimates of α_1_, α_*L*_, and σ_*L*_

(19) $$\begin{array}{l} r^{*}\;:=\;\frac{1-\exp\;\left(-\frac{PW}{\tau_{1}^{*}}\right)}{1-\exp\;\left(-\frac{PW}{\hat{\tau_{1}}}\right)}, \end{array}$$

we obtain $\alpha_{1}^{*}=r^{*}\hat{\alpha_{1}}$, $\alpha_{L}^{*}=r^{*}\hat{\alpha_{L}}$, and $\sigma_{L}^{*}=r^{*}\hat{\sigma_{L}}$ according to Equation (18). Given obtained $\hat{\theta }$, $\alpha_{1}^{*}$, $\alpha_{L}^{*}$, and $\sigma_{L}^{*}$ depend on r^*^. One can find that r^*^ is a decreasing function of $\tau_{1}^{*}\in \left(\tau_{1}^{l},\tau_{1}^{u}\right)$. Hence, one has r^*^ ∈ (*r*^*l*^, *r*^*u*^), where $r^{l}=\frac{1-\exp\;\left(-\frac{PW}{\tau_{1}^{u}}\right)}{1-\exp\;\left(-\frac{PW}{\hat{\tau_{1}}}\right)}$ and $r^{u}=\frac{1-\exp\;\left(-\frac{PW}{\tau_{1}^{l}}\right)}{1-\exp\;\left(-\frac{PW}{\hat{\tau_{1}}}\right)}$. Using the ratio between the boundaries of r^*^, one can find the interval for the parameter with set non-identifiability

(20) $$\begin{array}{l} r\;:=\;\frac{r^{u}}{r^{l}}=\frac{1-\exp\;\left(-\frac{PW}{\tau_{1}^{l}}\right)}{1-\exp\;\left(-\frac{PW}{\tau_{1}^{u}}\right)}. \end{array}$$

With *PW* = 0.42 ms, $\tau_{1}^{l}=10^{-2}$ ms, and $\tau_{1}^{u}=3$ ms in Table 2, we obtain *r* = 7.6545. However, we expect the ratio between the boundaries for τ_1_ is 3/0.01 = 300, i.e., non-identifiability spanning the entire range. To illustrate our theoretical analysis, we employ the profile likelihood approach to the elementary dataset from subject D9001 on Day 1 using the setting *TS*_2_. We expect flatness to exist in the *LPL* plot with respect to each of those four parameters. For α_1_, α_*L*_, and σ_*L*_, the flatness should only span a finite interval with the ratio of the range of 7.6545. So, for any positive value of τ_1_, we find that α_1_, α_*L*_, and σ_*L*_ are structurally non-identifiable due to a single value for PW for all stimuli. The *LPL* is flat over the entire parameter range. With a narrower range $\left[\tau_{1}^{l},\tau_{1}^{u}\right]$ for τ_1_, the *LPL* around the estimate could be flat over a smaller range.

#### 4.2.2. Illustrative examples with numerical results of profile-likelihood approach

Applying the PL approach, we present the possible types of parameter identifiability from the existing experimental datasets. First, to validate results of optimal fits and estimates obtained in Section 3, we perform the PL approach to the 15 elementary datasets on Day 1 using *TS*_1_. We compare PL results to the optimization results in Section 3. The PL approach gives identical estimates and optimal fit for 12 datasets. For the other three cases, we find negligible differences in optimal fits, i.e., $-2\;\log\left(\hat{L}\right)$, see details in the Supplementary Material. In addition, qualitatively the estimates are similar, i.e., estimates from the PL approach still stay on any boundary of **Θ** similar to those from the multiple-starting-value scheme. This validates our usage of datasets for both model comparison and identifiability analysis.

**Figure 4:**
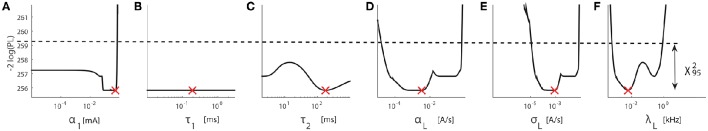
**Profile likelihood plot for the structurally non-identifiable case using the dataset containing about 200 data points using the setting ***TS***_**2**_ from subject D9001**. Red crosses indicate the estimated values of parameters from the multiple-starting value optimization. The horizontal dashed line marks 95% confidence intervals. Panels **(A–F)** correspond to six system parameters α_1_, τ_1_, τ_2_, α_*L*_, σ_*L*_, σ_*L*_, and λ_*L*_, respectively.

Second, to illustrate the structural non-identifiability, we show *LPL* for subject D9001 using the setting *TS*_2_ in Figure 4. The 95% confidence interval of each parameter is indicated by the region below the horizontal dashed line with the offset $\chi_{95}^{2}=3.84$, see Figures 4A–F. As we expect from Equation (18), the flatness of *LPL* at the minimal value occurs for parameters α_1_, τ_1_, α_*L*_, and σ_*L*_. For τ_1_, the flatness spans its entire range. On the other hand, for α_1_, α_*L*_, and σ_*L*_, the flatness of *LPL* at the minimum spans a shorter interval. The ratio between the upper and lower boundaries of this interval is about 7.6545. This agrees with the computed value from Equation (20).

**Figure 5:**
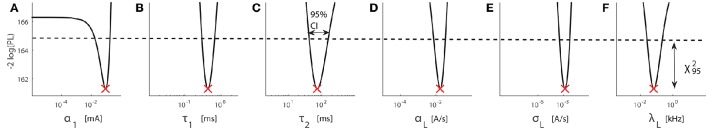
**Profile likelihood plot for subject D9450 with about 200 data points using the setting ***TS***_**1**_**. Red crosses indicate the estimated values of parameters from the multiple-starting value optimization. The horizontal dashed line marks 95% confidence intervals. Panels **(A–F)** correspond to six system parameters α_1_, τ_1_, τ_2_, α_*L*_, σ_*L*_, and λ_*L*_, respectively.

Third, for the identifiable case, we show −2 log(*PL*) for subject D9450, see Figure 5. The intersections of −2 log(*PL*) with the dashed horizontal line are the bounds of the 95% confidence interval. All six model parameters are practically identifiable at least up to the 95% confidence level. We have checked the difference between the empirical confidence interval and a χ^2^-distribution using a simulation study, see Section 3 in the Supplementary Material.

**Figure 6:**
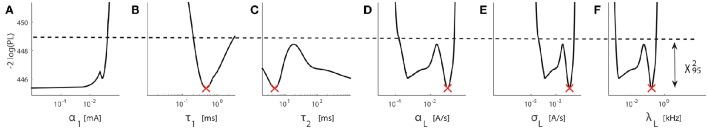
**Profile likelihood results with about 400 points measured from two-consecutive-day measurements on the subject D4443 using the setting ***TS***_**1**_**. Red crosses indicate the estimated values of parameters from the multiple-starting value optimization. The horizontal dashed line marks 95% confidence intervals. Panels **(A–F)** correspond to six system parameters α_1_, τ_1_, τ_2_, α_*L*_, σ_*L*_, and λ_*L*_, respectively.

Fourth, we give an example of practical non-identifiability with the combined dataset from subject D4443, see Figure 6. The *LPL* extends to the lower boundary of α_1_, indicating severe practical non-identifiability. Given the unbounded 95% confidence intervals of τ_2_, the time constant τ_2_ is also practically non-identifiable. In addition, we observe that profile likelihoods of the parameters α_*L*_, σ_*L*_, or λ_*L*_ have multiple local minima. Taken together, it indicates that the existing experimental data is insufficient to constrain model parameters. More measurements are required to further constrain the parameters toward narrower confidence intervals.

In addition to the results above for two datasets in Figures 5, 6, for the design of *TS*_1_, we summarize PL results for datasets with interior estimates in Supplementary Material. They consist of four elementary datasets and one combined dataset. We suspect that practical non-identifiability results from a limited amount of observations in the current 10 min detection task. As the PL-based parameter identifiability stems from noisy measurements, relatively noisy data hamper practical identifiability. For the five elementary datasets from Day 1 with interior estimates, detection probabilities from subject D9450 (Figure 2) exhibit a more monotone pattern with respect to amplitude than the other datasets (from D4523, D4543, D8846, and D9798). This may explain why the results for subject D9450 exhibit the best identifiability among the five. We notice that there is a case with severe practical non-identifiability for α_1_ with data from subject D4443, shown in Supplementary Figure 6A. In contrast to the stimulus set with *TS*_2_, we did not observe that a single parameter was severely non-identifiable for all the seven cases. This indicates that a stimulus set with all stimulus properties varied, e.g., *TS*_1_, may facilitate structural identifiability.

## 5. Discussion

In our study, we integrated experimental data with the hazard model for parameter estimation and identifiability of physiological parameters of human nociceptive processing. We obtained good fits with the HM to data according to statistics for goodness of fit. Based on BIC, comparison with the logistic regression model suggested a better balance between fit and complexity for the HM. By applying the profile likelihood approach, we demonstrated that it was possible to achieve parameter identifiability using 10 min measurements contains about 200 stimulus-response pairs, but not always. For structural identifiability, our theoretical analysis provided a necessary condition about the pulse width.

In conventional psychophysical studies, stimulus-response pairs are usually fitted to logistic and similar models (Treutwein, 1995). There are few studies to estimate physiology-based model parameters using psychophysical data, see e.g., Alcalá-Quintana and García-Pérez (2013). To the best of our knowledge, our study is the first to demonstrate the estimation of multiple model parameters characterizing peripheral and central nociceptive subsystems using binary responses to electrocutaneous stimuli. In addition, our study demonstrates the applicability of the PL approach with stimulus-response measurements and its merit to assess both structural and practical identifiability. As this study is a starting point for a mechanism-based diagnosis of the status of nociceptive systems, we have analyzed the hazard model of essential nociceptive subsystems, i.e., peripheral activation and processing by spinal neurons in the dorsal horn. In the present study about the HM, statistics from goodness of fit show reasonable results in general. However, this study did not account for other effects, e.g., non-stationarity of nociceptive processing as reported in Doll et al. (2014, 2016). On one hand, the non-stationary was not reported on individual level quantitatively. On the other hand, experimental properties like inter-stimuli intervals and intensities could play roles on presence of non-stationarity like habituation in a detection task (von Dincklage et al., 2013). A next step to explore neuronal or psychological mechanisms for possible non-stationarity in nociceptive processing could be to extend the hazard model with time-varying physiological parameters in peripheral and central subsystems, i.e., α_1_ and α_*L*_, respectively. With such extended models, one may perform model comparison with the HM to speculate time-varying processes to contribute to observed non-stationary effects.

In this study, we compared the BIC-values from the two models using datasets with *TS*_1_. Although, in 24 out of 30 cases, the HM had smaller BICs than those from the logistic regression model, we notice this may be attributed to the difference in the model complexity rather than the difference in model fits, see Figure 3. This indicates that the fit by the logistic regression model to data is not worse than that from the HM, although the logistic regression model does not explicitly represent nociceptive mechanisms. Our present study did not present the BIC of the HM using a group of datasets with *TS*_2_. Given structural non-identifiability shown in Figure 4, via model reduction by fixing τ_1_ to a constant value, one can expect that the reduced model would still have the same optimal fit $-2\;\log\left(\hat{L}\right)$ as the six-parameter hazard model. For the (reduced) HM, the BIC-value will further decrease, leading to a better balance between model fit and complexity. In our present study, only for the design *TS*_2_, we provided an analytically derived assessment of parameter identifiability in the HM together with an illustrative example with numerical simulations. The present study did not answer which combinations of temporal properties will achieve optimal identifiability for parameters at an arbitrary value. This is partly challenged by the non-linearity of the physiology-based model, for which pure simulations with one set of parameter values might not be comprehensive. So, a future study with both analytical derivation and simulations might be useful to get thorough understanding of the effects of combinations of temporal properties. Future work could consider the choice of stimulus properties in order to resolve practical non-identifiability in a model-based study. In addition, one could choose a frequentist (Steiert et al., 2012) or adapt a Bayesian framework (Myung et al., 2013) in the exploration of stimulus settings.

Typically, for dynamical models representing biochemical processes, PL plots of structurally non-identifiable parameters were flat for the entire interval, which is restricted by pre-defined lower and upper boundaries (Raue et al., 2009). In our identifiability analysis of the hazard model with six parameters, first, we show that a stimulus set with invariant *PW* results in four structurally non-identifiable parameters α_1_, τ_1_, α_*L*_, and σ_*L*_. Second, we formulated the ratio *r* in Equation (20) to quantify the range of set identifiability in an analytically tractable way. This ratio depends on the boundaries of τ_1_ and temporal property *PW*. Its upper boundary has a larger impact on the ratio than the lower one. On the other hand, when the *PW* is not varied in the stimulus set, the larger *PW* is, the smaller *r* is, see Appendix B. It implies that saturation of activation of Aδ fibers helps to distinguish different nociceptive mechanisms with a smaller interval of set identifiability for non-identifiable α_1_, α_*L*_, and σ_*L*_. Together with prevention of repetitive recruitment of the same nerve endings during one pulse, we suggest to use a value of *PW* like 1.05 ms. In addition to these analytical arguments, the PL approach can further determine the boundary values of set identifiability for α_1_, α_*L*_, and σ_*L*_ given experimental datasets, see Figures 4A,D,E.

Mechanism-based diagnosis of (mal)functioning of the nociceptive system may benefit from our developed approach on parameter estimation and identifiability analysis. In pain research, various experimental pain models have been developed to perturb the nociceptive system (Szallasi, 2010). For example, high frequency electrical stimulation is known to induce central sensitization (Sandkühler, 2009). Further validation studies would be useful to test our quantitative approach with psychophysical datasets measured from sequential phases in experimental pain models. In clinical practice, diseases or medical intervention could result in neuroplasticity, accompanying with (mal)adaptive behavior. With our methods, in turn, new insights on responsive mechanisms underlying nociceptive malfunctioning could be gained by monitoring changes in estimates of parameters over time.

## Author contributions

HY, HM, JB, and SG designed and conceived the study. HY and HM performed the mathematical analysis. HY drafted the manuscript. HM, JB, and SG commented and edited the manuscript. All authors read and approved the final manuscript.

### Conflict of interest statement

The authors declare that the research was conducted in the absence of any commercial or financial relationships that could be construed as a potential conflict of interest.

## Appendix

### Appendix A: computation of profile likelihood

Here, we give the details on the computation of profile likelihood. Given the estimate **θ**, we compute the profile likelihood according to Equation (14). For each single parameter **θ**_*i*_, we divide the computation for the entire parameter range into two sub-interval by the estimate **θ**_*i*_. For each sub-interval, we use an adaptive method to determine step Δ_θ_. For illustrative purposes, we explore values of the parameter $\theta_{i}^{\left(n\right)}$ in the increasing direction with respect to $\hat{\theta_{i}}$,

(A1) $$\begin{array}{l} \theta_{i}^{\left(n\right)}=\theta_{i}^{\left(n-1\right)}+\Delta_{\theta },\;n=1,2,\ldots ,500, \end{array}$$

where $\theta_{i}^{\left(0\right)}:=\hat{\theta_{i}}$. The step Δ_θ_ could be solved from the univariate equation about Δ_θ_: $-2\;\log\left(L\left(\theta_{i}^{\left(n\right)}\right)\right)=-2\;\log\left(L\left(\theta_{i}^{\left(n-1\right)}\right)\right)+q\chi_{\alpha }^{2}$, where by default we set *q* = 0.05. If this equation is not solvable, we set $\theta_{i}^{\left(n\right)}=1.05\theta_{i}^{\left(n-1\right)}$. Given the value of $\theta_{i}^{\left(n\right)}$, the profile likelihood in Equation (14) is determined by re-optimizing values of remaining parameters **θ**_{/*i*}_.

### Appendix B: the range of the set identifiability is a decreasing function of the *PW*

We have

(A2) $$\begin{array}{l} r=\frac{1-\exp\;\left(-\frac{PW}{\tau_{1}^{l}}\right)}{1-\exp\;\left(-\frac{PW}{\tau_{1}^{u}}\right)}. \end{array}$$

The derivative of *r* with respect to *PW* is given by

(A3) $$\begin{array}{l} \frac{dr}{dPW}=\frac{\exp\;\left(PW\left(\frac{1}{\tau_{1}^{u}}-\frac{1}{\tau_{1}^{l}}\right)\right)\;(\tau_{1}^{l}\left(1-\exp\;\left(PW/\tau_{1}^{l}\right)\right)+\tau_{1}^{u}\;(\exp\;\left(PW/\tau_{1}^{u}\right)-1))}{\tau_{1}^{l}\tau_{1}^{u}{\left(\exp\;\left(PW/\tau_{1}^{u}\right)-1\right)}^{2}} \end{array}$$

Given $\tau_{1}^{l}<\tau_{1}^{u}$, the sign of $\frac{dr}{dPW}$ is equal to the sign of

(A4) $$\begin{array}{l} r_{1}:=\tau_{1}^{l}\left(1-\exp\;\left(PW/\tau_{1}^{l}\right)\right)+\tau_{1}^{u}\left(-1+\exp\;\left(PW/\tau_{1}^{u}\right)\right). \end{array}$$

The derivative of *r*_1_ with respect to *PW* is $\frac{dr_{1}}{dPW}=\exp\left(PW/\tau_{1}^{u}\right)-\exp\left(PW/\tau_{1}^{l}\right)<0$, indicating that *r*_1_ decreases as *PW* increases. For *PW* = 0, we have *r*_1_ = 0, so *r*_1_ is negative for positive *PW*, and $\frac{dr_{1}}{dPW}<0$ as well. So the quantity *r* is a deceasing function of *PW* according to Equation (A3).
