# Supplementary material for: Estimation and Identifiability of Model Parameters in Human Nociceptive Processing Using Yes-No Detection Responses to Electrocutaneous Stimulation
Source: Front Psychol. 2016 Dec 5;7:1884. doi: 10.3389/fpsyg.2016.01884 (PMC5136566; doi:10.3389/fpsyg.2016.01884)
Supplement: Supplementary file 1 [file DataSheet1.PDF]

Supplementary Material: Estimation and identifiability of model parameters in human nociceptive processing using yes-or-no detection responses to electrocutaneous stimulation

H. Yang*, H.G.E. Meijer, J.R. Buitenweg and S.A. van Gils
*Correspondence:
Huan Yang
h.yang-1@alumnus.utwente.nl

First, we show the performance of fitted psychometric curves by the hazard model (HM) to 30 datasets. Second, we show optimization performance of MLE and PL. Third, we perform a validation study to check the profile likelihood (PL) approach, i.e. whether a χ^2-distribution-based threshold is valid to determine a confidence interval. Fourth, we show how the PL approach performs for several experimental datasets. Fifth, for set identifiability, we show dependence between parameters along the parameter manifold. Lastly, we check the effects of (i) number of datapoints, (ii) combinations of temporal properties and (iii) possible non-stationary psychometric curves on the estimation performance. We use the bootstrapping approach to compare with the profile likelihood approach.

1 FITTING PERFORMANCE OF MODELS TO EXPERIMENTAL DATASETS

We consider the 30 elementary datasets from the detection task described in section 2.1. Each dataset contains about 200 stimuli and binary responses. Applying the multiple-starting value optimization, we obtain an optimal fit of the model for each dataset. In Figs. S1 and S2, we show optimal fits of the HM (red) and the logistic regression model (blue) to these datasets. Rows A-D correspond to the four combinations A-D of temporal properties of TS_1. In each figure, the columns indicate 15 different subjects.

For the datasets from subjects D9450 and D4443 (measured on Day 1), we obtained the likelihood for 100 starting values. Ranking the runs by increasing likelihood reveals a step-like pattern as in (Raue et al., 2013), see Fig. S3. This indicates that our local optimization is efficient.

2 OPTIMAL MODEL FITS AND PARAMETER ESTIMATES

Here we summarize the estimates of six parameters for all 30 elementary datasets. Supplementary Table S1 and S2 list parameter estimates for data measured on Day 1 and Day 2 from the multiple-starting-value optimization. In addition, for data from Day 1, we compare the estimates and optimal fits from the PL approach to those using the multiple-starting-value optimization. Comparison shows that there are three cases where even better model fits are found. However, the difference among the three paired comparisons of $-2\log(L)$ is always smaller than 0.04. Also, these estimates are often very close in two cases. Hence, in this more extensive exploration of the parameter space, no significantly better model fits are found.

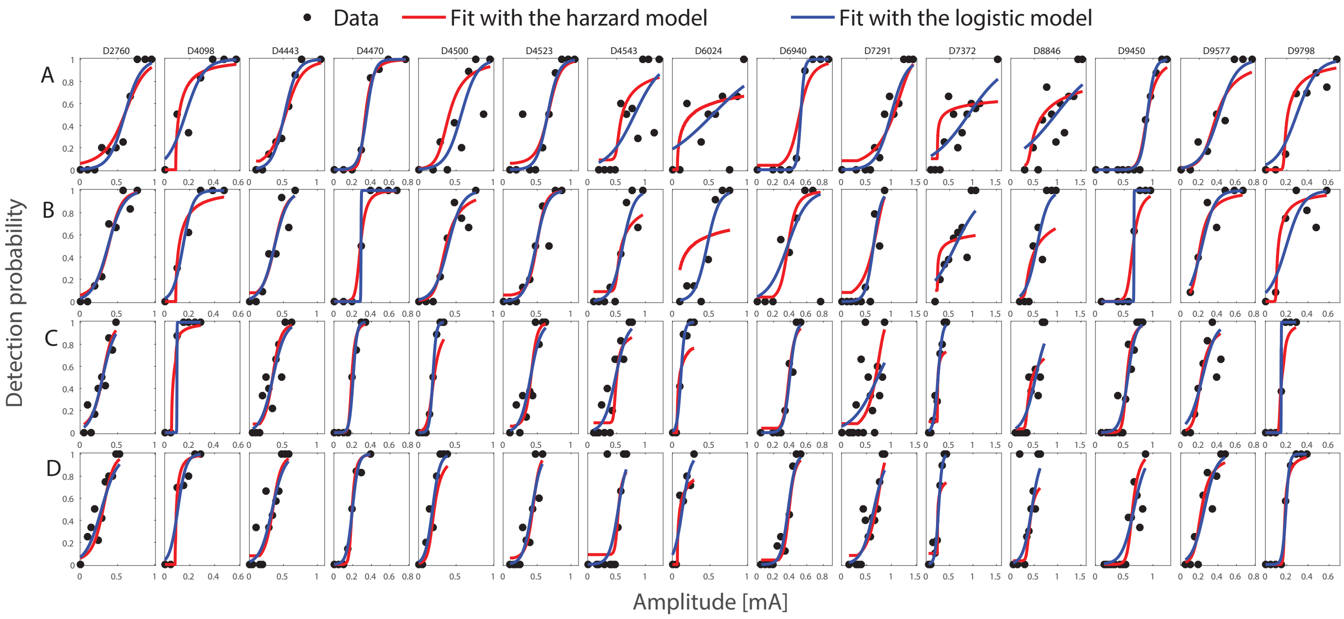

Supplementary Figure 1. Fitting performance of the HM and the logistic model to the experimental stimulus-response pairs measured on Day 1.

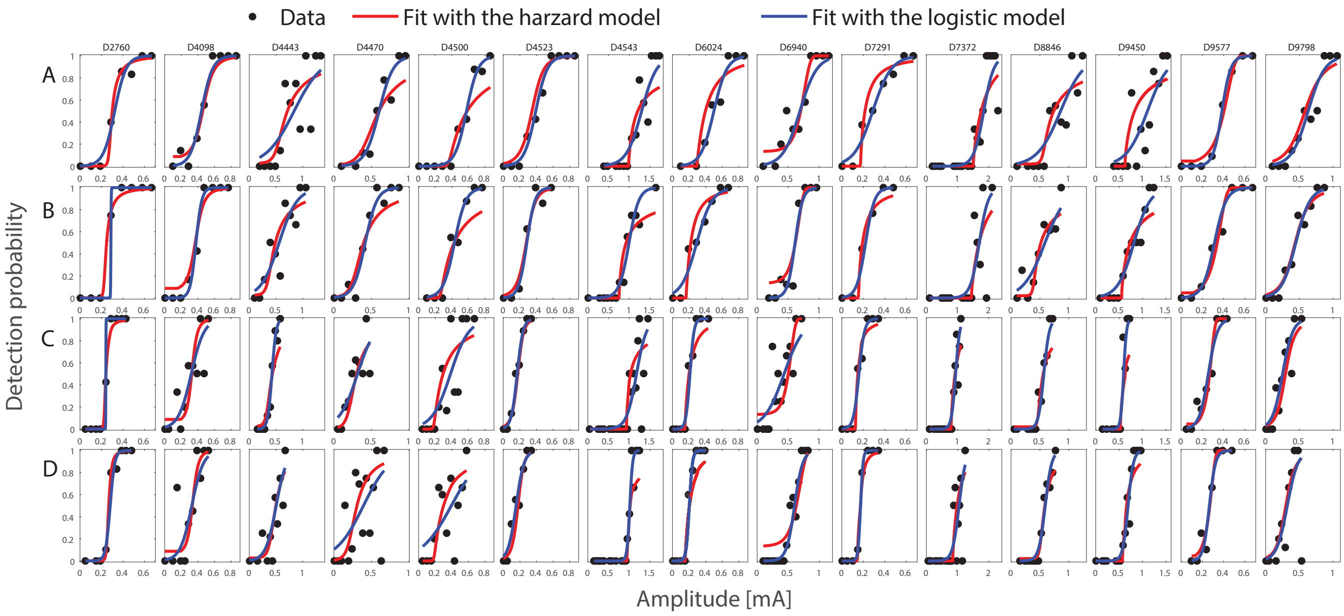

Supplementary Figure 2. Fitting performance of the HM and the logistic model to the experimental stimulus-response pairs measured on Day 2.

This suggests that $N_s = 100$ for the multiple-starting-value optimization is sufficient. We also performed a goodness-of-fit test by using likelihood-ratio test. We computed the ratio of the likelihood from the saturated model (i.e. detection probabilities based on binomial fit at each amplitude) and the presented HM. We reported both G^2-statistics (i.e. the ratio of the likelihoods) and their p value from a χ^2-distribution with corresponding degree of freedom. For a significance level of $\alpha = 0.05$, we highlight the rejected models with p value in bold. Only 2 out of 30 cases have relatively low p values. We conclude that our HM has a reasonable fit to the measured datasets.

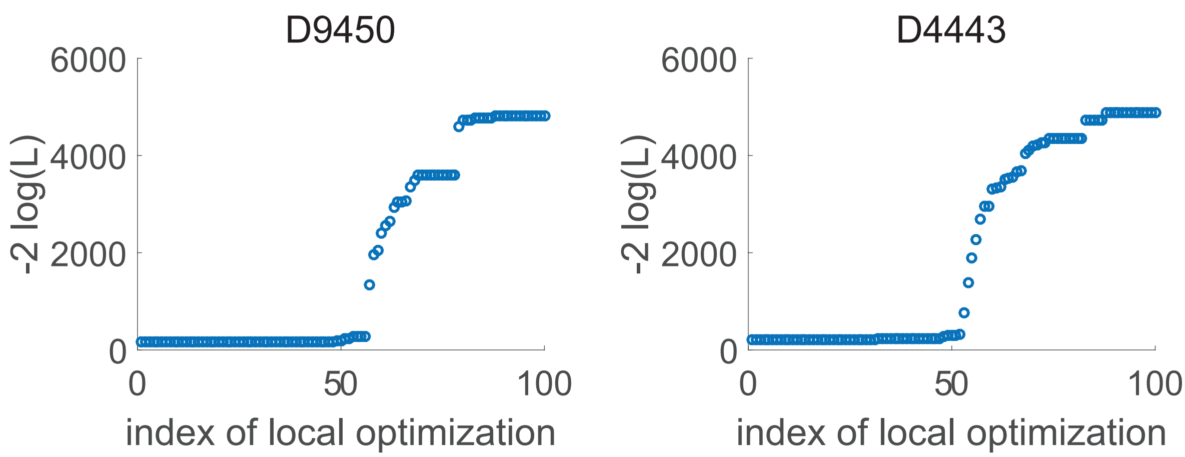

Supplementary Figure 3. Estimates sorted by increasing $-\log(L)$. The steps indicate jumps between global and local minima in the estimation.

Supplementary Table 1. Parameter estimates and optimal fits for 15 elementary datasets using TS_1 measured on Day 1 using the multiple-starting-value method. We perform profile likelihood method, detecting three cases with different estimates and fits. The differences are marked in red. The likelihood ratio test statistics and corresponding p value are shown for each datasets, see G^2 and p, respectively.

Model parameter	α_1 [mA]	τ_1 [ms]	τ_2 [ms]	α_L [A/s]	σ_L[A/s]	λ_L [kHz]	$-2\log(\hat{L})$	G^2	p
D2760	0.000001	0.75	649.9	0.0014	0.000241	0.0096	201.33	19.37	0.985
D4098	0.000573	0.04	9.8	0.0194	0.000000	0.1215	103.86	19.86	0.530
	0.000004	0.01	17.6	0.0124	0.000000	0.0802	103.83		
D4443	0.083333	0.51	1000.0	0.0008	0.000163	0.0042	219.13	32.87	0.618
D4470	0.033727	0.26	1000.0	0.0007	0.000058	0.0052	98.41	8.52	1.000
D4500	0.000001	0.20	1000.0	0.0009	0.000146	0.0025	174.21	42.70	0.062
D4523	0.095231	0.40	166.0	0.0068	0.000935	0.0438	162.85	29.39	0.809
D4543	0.368583	0.11	16.6	0.0200	0.002899	0.0470	192.49	63.54	**0.016**
D6024	0.001621	0.02	4.3	0.0265	0.000001	0.0694	185.89	42.13	**0.024**
	0.001590	0.02	4.3	0.0265	0.000000	0.0694	185.86		
D6940	0.161138	0.43	1000.0	0.0005	0.000096	0.0034	166.32	41.97	0.112
D7291	0.115707	0.93	1000.0	0.0032	0.000219	99.9803	202.85	48.05	0.471
D7372	0.230783	0.01	2.0	0.0050	0.000713	0.0586	223.15	30.33	0.693
D8846	0.194096	0.28	67.0	0.0047	0.000771	0.0076	227.76	36.92	0.653
D9450	0.093299	0.44	72.9	0.0174	0.001357	0.0677	161.31	32.67	0.896
D9577	0.006398	3.00	1000.0	0.0001	0.000021	0.0021	176.13	25.97	0.627
D9798	0.006116	1.38	9.2	0.0097	0.000368	0.1427	119.37	18.06	0.840
	0.006109	1.38	9.2	0.0097	0.000368	0.1428	119.37		

3 VALIDATION OF PROFILE LIKELIHOOD APPROACH

We check whether it is valid to use a χ^2-distribution to assign a threshold for the confidence interval (e.g. we set the threshold χ^2_α=3.84 to determine the CI at significant level of $\alpha = 0.95$). For one parameter θ_k with $k = 1, 2, ..., 6$, we proceed as follows similarly to the approach employed by (Kreutz et al., 2012). Suppose with multiple replicates of measurements and known true values of parameters, we need to collect the set for the thresholds to determine confidence intervals. Here, we treat the estimate $\boldsymbol{\theta}^*$ as the true parameter values, and use all stimulus properties $\{S\}$ from the existing dataset, $Data$. We generate 150 datasets using the HM and the set of stimuli $\{S\}$. Hence, each generated dataset has the same amount of datapoints as $Data$.

for a noise realization i **do**

- Generate a dataset $Data_i$ with yes-no responses $\{R\}$ to $\{S\}$ using the hazard model with $\boldsymbol{\theta}^*$.

Supplementary Table 2. Parameter estimates and optimal fits for 15 elementary datasets using TS_1 measured on Day 2 using the multiple-starting-value method.

Model parameter	α_1 [mA]	τ_1 [ms]	τ_2 [ms]	α_L [A/s]	σ_L[A/s]	λ_L [kHz]	$-2\log(\hat{L})$	G^2	p
D2760	0.132516	0.27	38.2	0.0062	0.000533	0.0609	76.34	6.70	1.000
D4098	0.143050	0.30	285.5	0.0023	0.000396	0.0157	161.70	38.93	0.257
D4443	0.065499	0.55	63.0	0.0104	0.001392	0.0238	190.21	35.42	0.634
D4470	0.000001	0.65	1000.0	0.0006	0.000106	0.0018	224.35	46.16	0.171
D4500	0.000001	0.42	1000.0	0.0007	0.000031	0.0014	188.11	48.29	0.101
D4523	0.000001	0.31	1000.0	0.0009	0.000138	0.0061	149.26	19.00	0.870
D4543	0.603967	0.35	84.0	0.0027	0.000000	0.0070	184.21	36.46	0.997
D6024	0.000001	1.98	1000.0	0.0002	0.000000	0.0023	153.29	27.04	0.516
D6940	0.000001	0.21	8.2	0.2905	0.020524	100.000	192.16	34.84	0.837
D7291	0.062048	0.20	51.4	0.0050	0.000085	0.0312	125.72	14.37	0.938
D7372	0.052303	0.13	259.7	0.0163	0.000000	0.0143	156.32	44.95	0.966
D8846	0.206076	0.50	50.3	0.0064	0.000862	0.0173	196.52	22.05	0.991
D9450	0.355383	0.21	45.0	0.0118	0.000000	0.0195	188.95	41.76	0.651
D9577	0.105230	0.25	1000.0	0.0023	0.000151	100.000	131.10	13.28	0.987
D9798	0.000001	0.39	1000.0	0.0012	0.000212	0.0039	209.46	30.19	0.779

- Compute the negative log-maximal likelihood LL_i with $Data_i$ and profile likelihood for parameter θ_k.
- Compute the difference $\delta_i = LPL(\theta_k = \theta_k^*) - 2LL_i$.

end for

To check the validity of the PL, we compare the empirical cumulative distribution function (ECDF) of $\{\delta_i\}$, with $i = 1, 2, ..., 150$ to the ECDF of χ^2 with 1 degree of freedom.

Here, we apply the above algorithm for all six parameters using two datasets of subjects D9450 and D8846 (measured on Day 1). From the ECDF, one can obtain the threshold to indicate the threshold for the

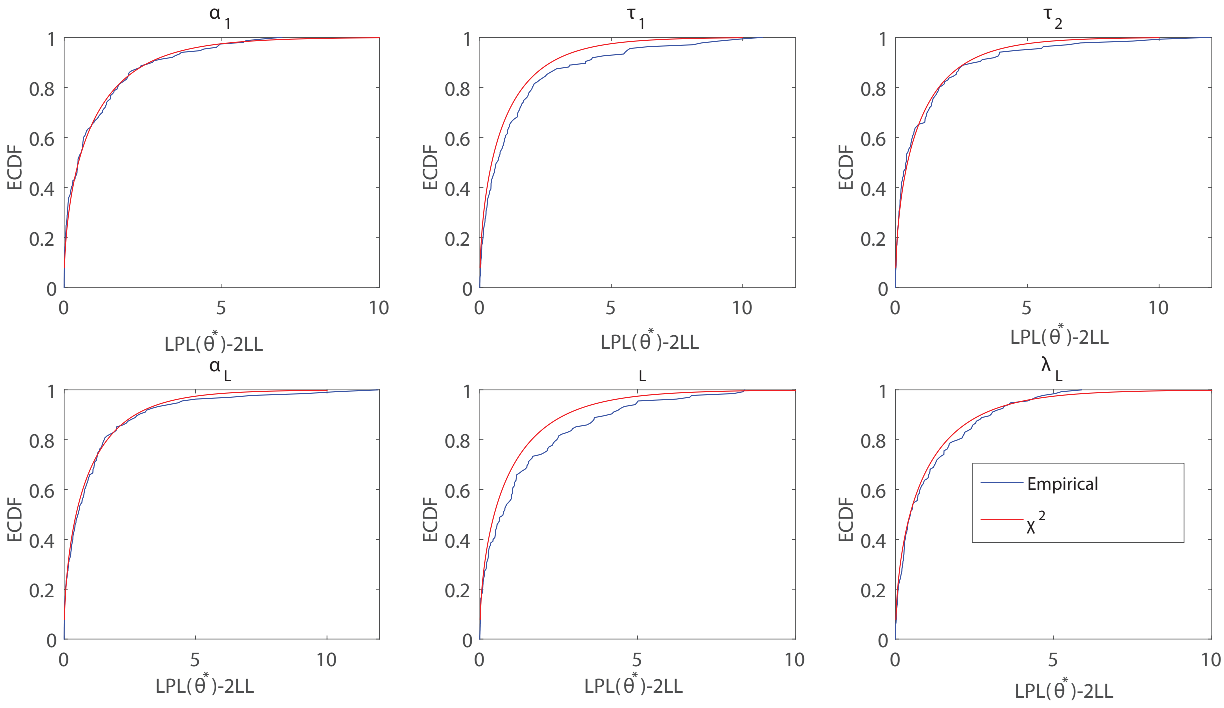

Supplementary Figure 4. Check the validity of coverage of true values of parameters in the PL results for subject D9450.

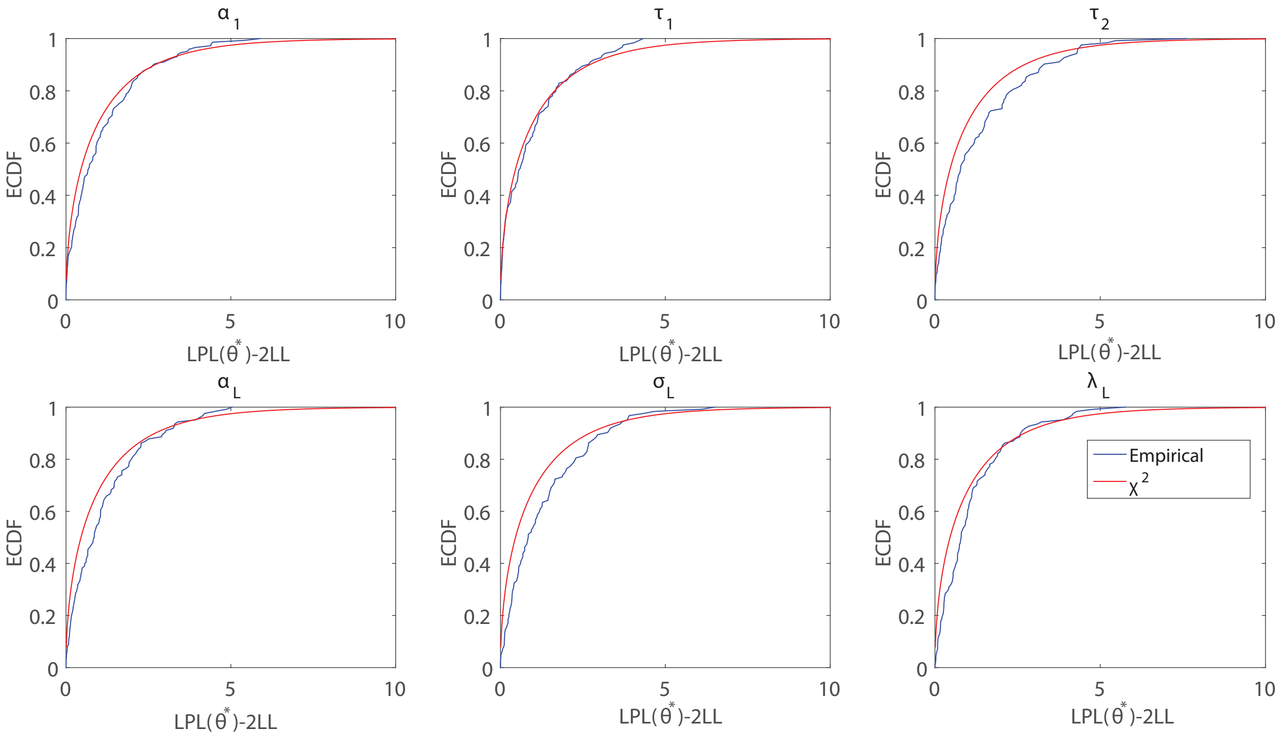

Supplementary Figure 5. Check the validity of coverage of true values of parameters in the PL results for subject D8846.

PL with a confidence interval at α level. The χ^2 distributions are similar to the empirical ones in general. We detect larger differences for parameter σ_L for both subjects than other parameters. Compared to results for D8846, the results for D9450 have a better approximation. The matlab scripts are uploaded to the folder `Parameter_estimation_Profile_likelihood` in supplementary material 2.

As another way to check the validity and robustness of the PL approach, we split the datasets into two parts: one with only measurements at odd trials, other with only measurements at even trials. Then, we computed the MLE and the profile likelihood for each split dataset. The PL results are shown in the Fig. S6. The overlap in each pair of PL plots shows rather consistent results for all system parameters (except the

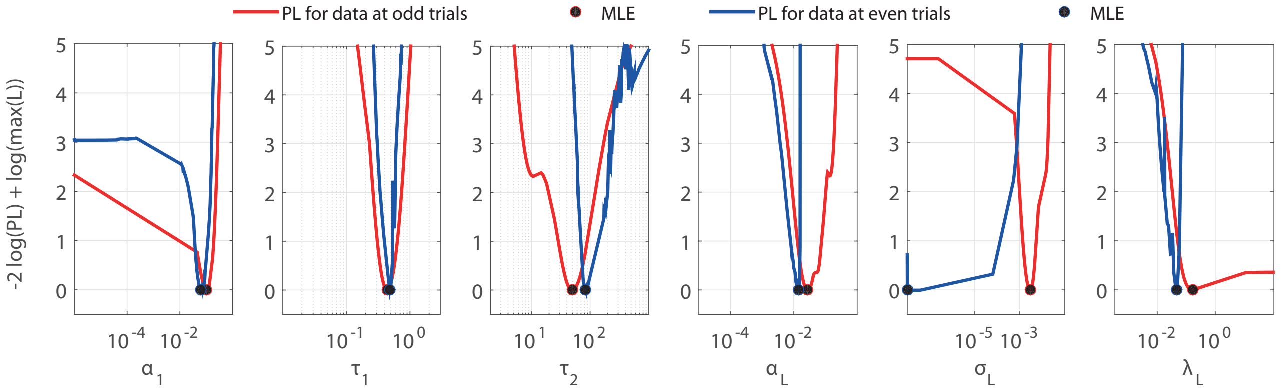

Supplementary Figure 6. MLEs and PLs for split datasets with odd or even-index measurements

noisy parameter σ_L).

4 PROFILE LIKELIHOOD RESULTS WITH INTERIOR ESTIMATES FOR EXPERIMENTS USING TS_1

Within the 15 elementary datasets on Day 1 using TS_1, there are 5 sets of SRPs yielding interior estimates of $\boldsymbol{\theta}$: for subjects D4523, D4543, D8846, D9450, D9798. For subjects D2760 and D4443, the profile likelihood was also performed to investigate parameter identifiability. We have already shown the profile likelihood for D9450 and D4443 in Figs. 5 and 6 in the manuscript, respectively. For the remaining five cases, we show the profile likelihood in Supplementary Figs. S7-S11.

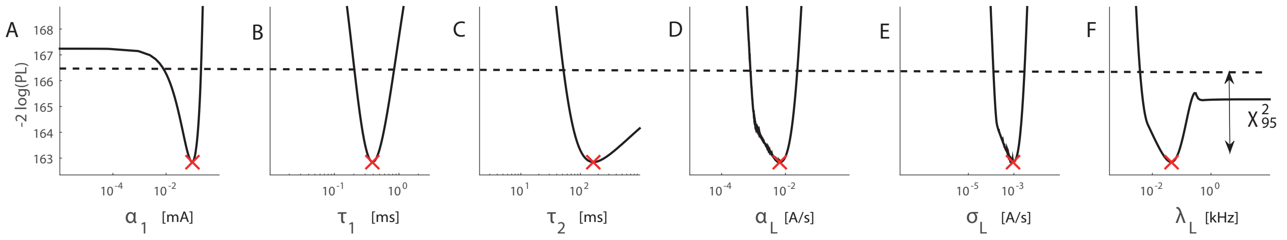

Supplementary Figure 7. PL results for the elementary dataset from subject D4523 measured on Day 1.

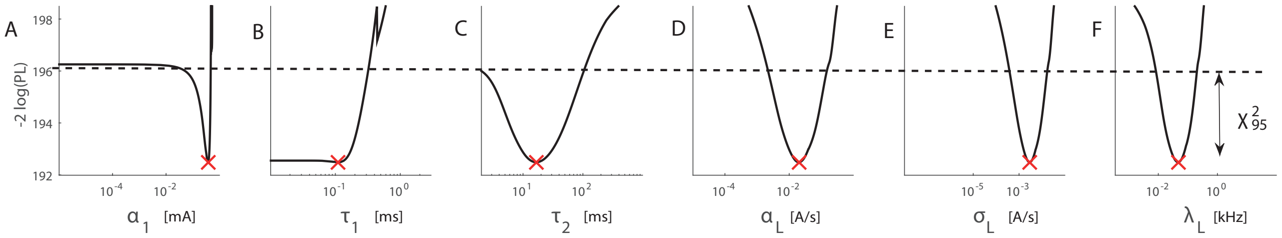

Supplementary Figure 8. PL results for the elementary dataset from subject D4543 measured on Day 1.

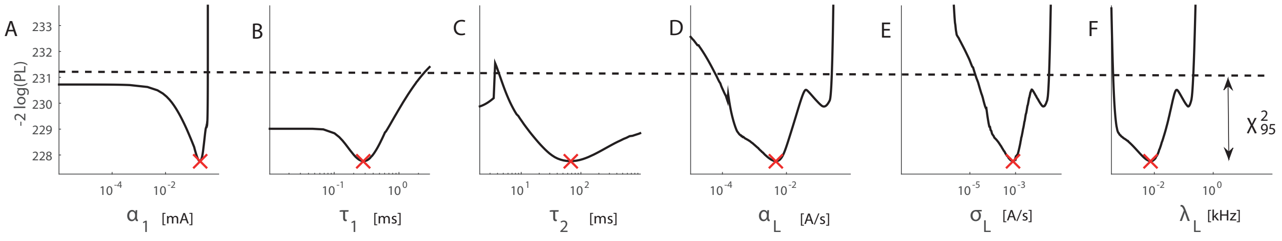

Supplementary Figure 9. PL results for the elementary dataset from subject D8846 measured on Day 1.

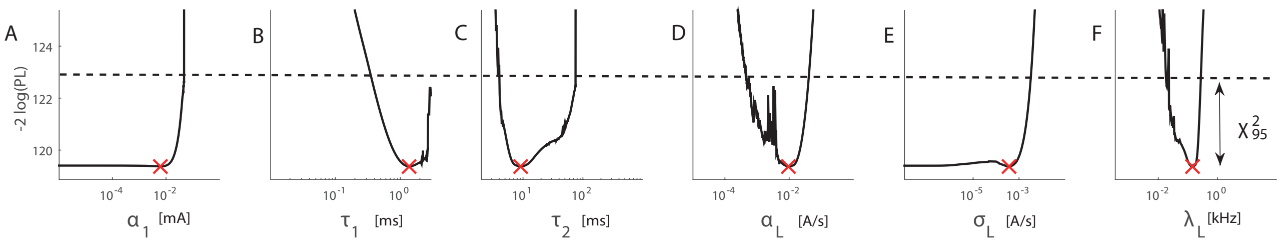

Supplementary Figure 10. PL results for the elementary dataset from subject D9798 measured on Day 1.

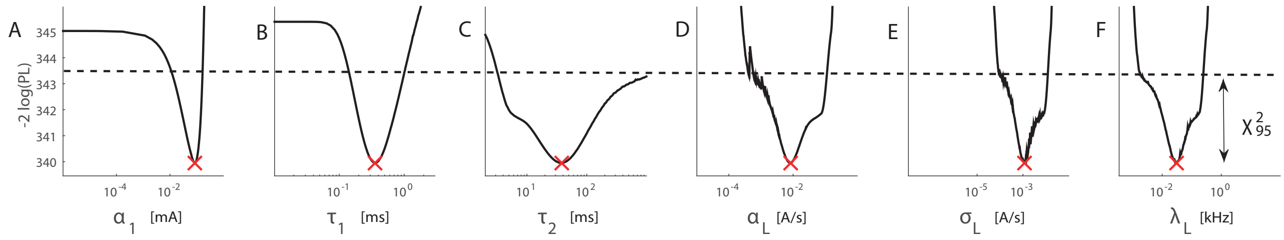

Supplementary Figure 11. PL results for the combined dataset from subject D2760 measured on Day 1 and 2.

5 PARAMETER DEPENDENCE ALONG THE SET-IDENTIFIABILITY

Using the setting TS_2, we study dependence of α_1^*, α_L^*, and σ_L^* on $\tau_1^* \in (0.01, 3)$. Here two different ways are employed and compared: (i) analytically derived expression (see Eq. (18 and 19) in the main text) and (ii) numerically implemented PL approach. In the latter, we obtained values of α_1^*, α_L^*, and σ_L^*, when we apply the PL approach to parameter τ_1. Fig. S12 shows similar relationships from both approaches.

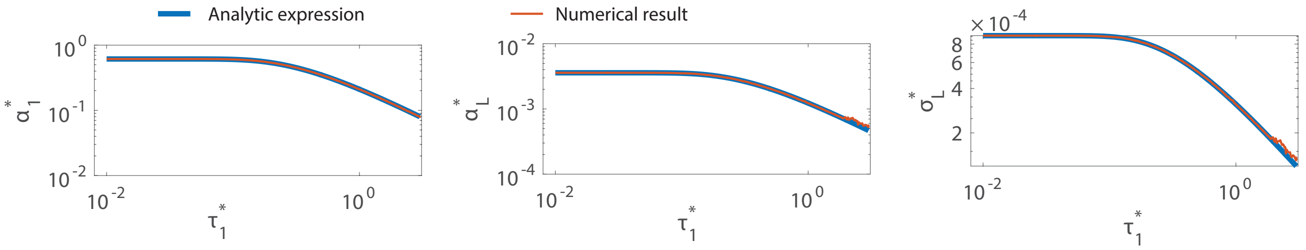

Supplementary Figure 12. Parameter dependence between parameters along the set identifiability with both analytical expression and the numerical evaluation of PL.

6 ADDITIONAL SIMULATIONS

6.1 Effect of the number of datapoints on estimation performance

We employ a non-parametric bootstrapping procedure to generate datasets. To generate one dataset, for each of four combinations of temporal properties, we randomly choose n_r datapoints (i.e. pairs of stimulus and response) from the existing dataset, where $n_r = 25, 35$, and 45. Then, we combine the sample data across different temporal combination to form the newly “generated” dataset. We repeat the generation steps to obtain multiple datasets.

For each dataset, we performed parameter estimation and computed the profile likelihood for each system parameter. The PL approach yields a conference interval, as we described. Qualitatively, the shape of the PL plot can inform us about the performance of estimation. In addition, with multiple datasets, one can check the estimation performance by looking at the statistics of a set of MLEs. We refer to this approach as the sampled-MLEs-based approach. We compare these two approaches, to evaluate their sensitivity to detect the effect of the number of datapoints on estimation performance.

When increasing the number of datapoints, one can expect a better estimation performance with a narrower confidence interval. For the PL approach, the PL plot should have a smaller width. For the sampled-MLEs-based approach, one would expect a more dense scatter plot of MLEs.

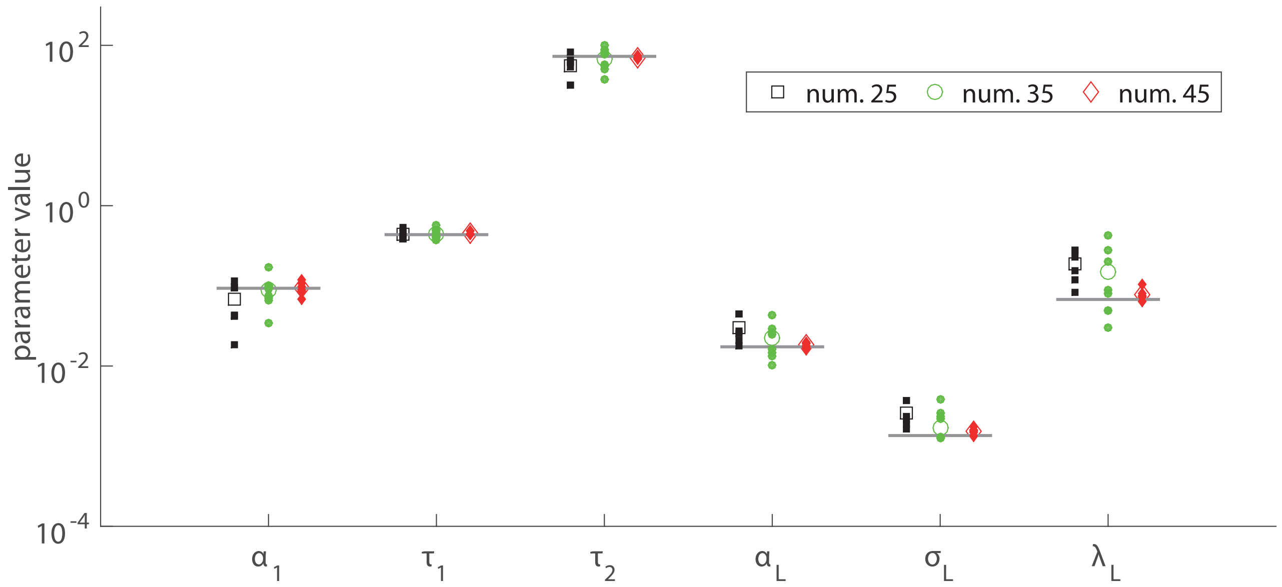

Supplementary Figure 13. Scatter plots of the estimates that are within the parameter domain. The big markers indicate the mean value for each parameter. Three numbers of datapoints are considered here: 4-by-25, 4-by-35 and 4-by-45.

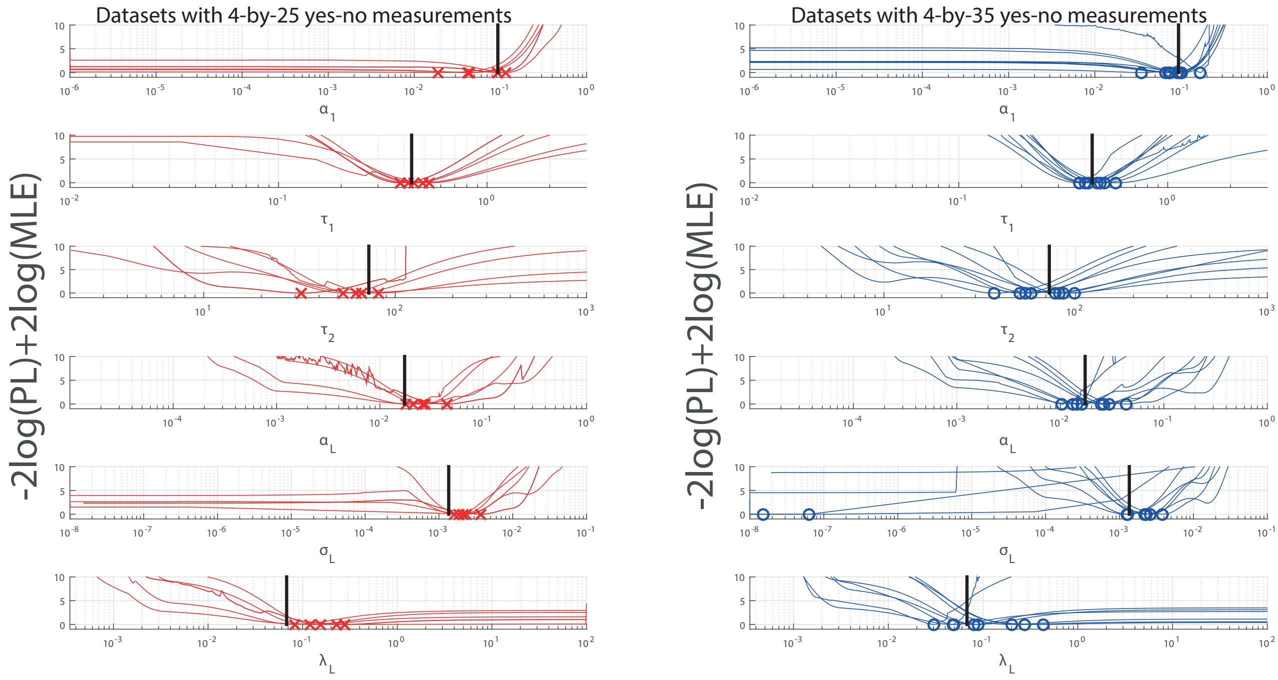

Supplementary Figure 14. MLEs and PLs for two split datasets with odd or even-index measurements

Supplementary Fig. S13 presents the scatter plot of MLEs from datasets sampled from a non-parametric bootstrapping approach. For $n_r = 45$, we have the narrowest distributed scatter plots of MLEs, see Supplementary Fig. S13. However, the scatter plots between the cases of 25 and 35 are similar. Using the profile likelihood approach, the effect of the number of data points (between these two groups with 4-by-25 or 4-by-35) are reflected in the shapes of PL (e.g. α_1 and τ_1), shown in Supplementary Fig. S14. Hence, the PL approach is sensitive to the number of data points used. This demonstrates the advantage of the latter one, which also agrees with results from a previous simulation study (Fröhlich et al., 2014).

6.2 Effect of temporal combinations

We consider five designs of the temporal properties. The first one is TS_1 with four combinations of temporal properties: (1) $(NoP = 1, PW = 0.42)$, (2) $(NoP = 1, PW = 0.84)$, (3) $(NoP = 2, IPI = 10, PW = 0.42)$, and (4) $(NoP = 2, IPI = 50, PW = 0.42)$. Each of the other four designs contains the subset with three combinations. We denote $TS_{\{/i\}}$ with $i = 1, 2, 3, 4$ to the design when we discard one combination i.

For each design of temporal properties, we employ a Monte-Carlo scheme to generate datasets by simulating the HM with estimated values ($\alpha_1 = 0.093299, \tau_1 = 0.44, \tau_2 = 72.9, \alpha_L = 0.0174, \sigma_L = 0.001357, \lambda_L = 0.0677$) for subject D9450. For each dataset, we perform parameter estimation and computed the profile likelihood for each system parameter. We present PL results for five designs TS_1, $TS_{\{/1\}}$, $TS_{\{/2\}}$, $TS_{\{/3\}}$, $TS_{\{/4\}}$ in Figs. S15,S16,S17,S18,S19, respectively.

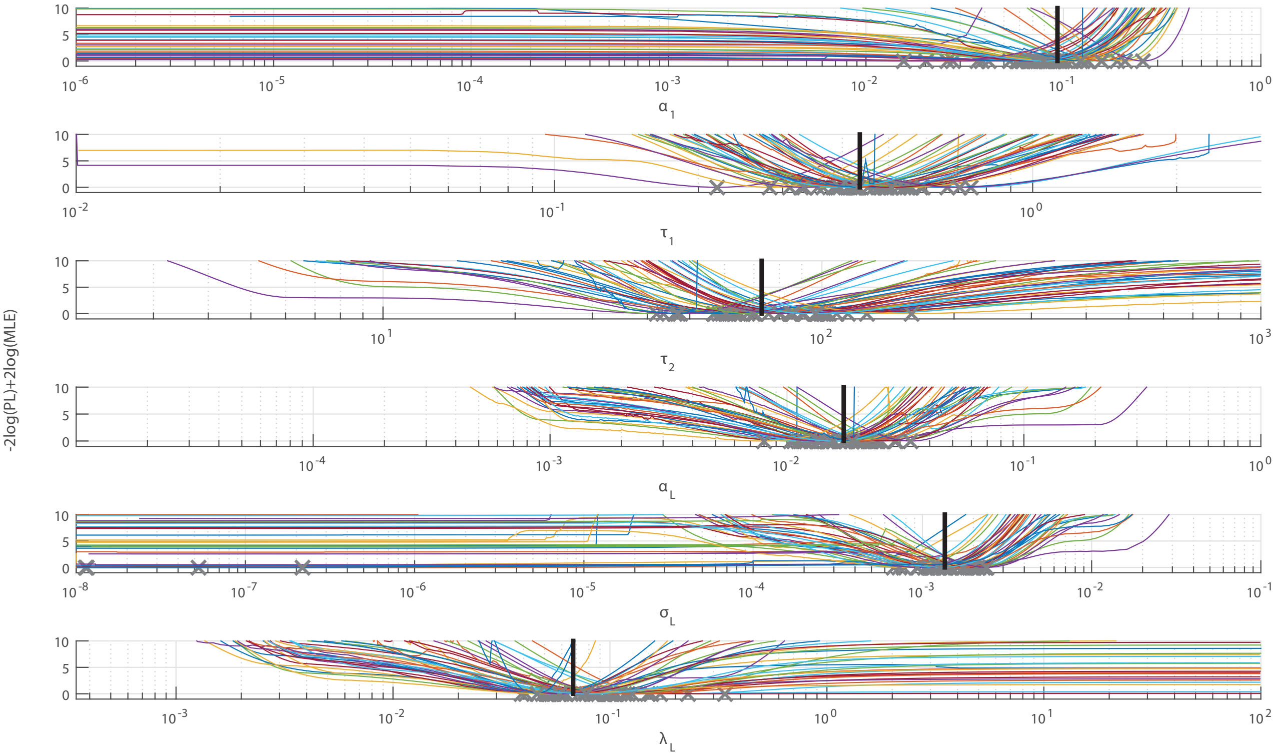

Supplementary Figure 15. PL results for the design of combinations of temporal properties TS_1

We present the scatter plots of MLEs with five designs of temporal properties in Fig. S20. However, results of scatter plots with different designs of combinations are rather similar to each other. Here, we highlight that the PL approach provides some extra information of the effects of temporal properties on the estimation performance.Using the design of temporal properties $TS_{\{/2\}}$, one can expect set-non-identifiability of parameters (α_1, α_L and σ_L) and non-identifiability for τ_1. However, for identifiability about parameter λ_L and τ_2, this design showed a better performance than their counterparts with three combinations. Also, its performance is comparable to that from design TS_1. In addition, using other designs $TS_{\{/1\}}$, $TS_{\{/3\}}$ or $TS_{\{/4\}}$, α_1 is always seriously non-identifiable in a practical sense. By comparison to Fig. S15, the design TS_1 with four combinations is recommended to prevent this.

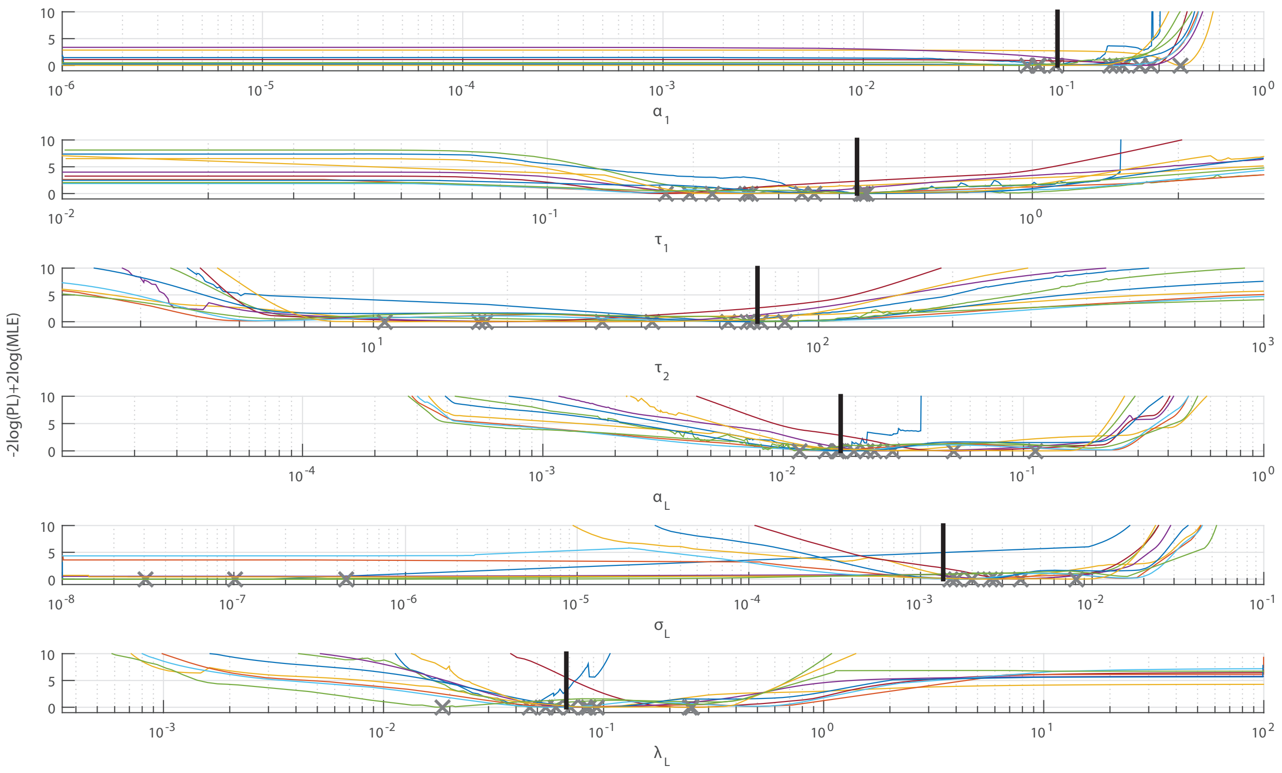

Supplementary Figure 16. PL results for the design of combinations of temporal properties $TS_{\{/1\}}$

110 This gives some feasibility to choose the combinations of temporal properties to more specifically
111 estimate different parameters, when experiments have a time restriction.

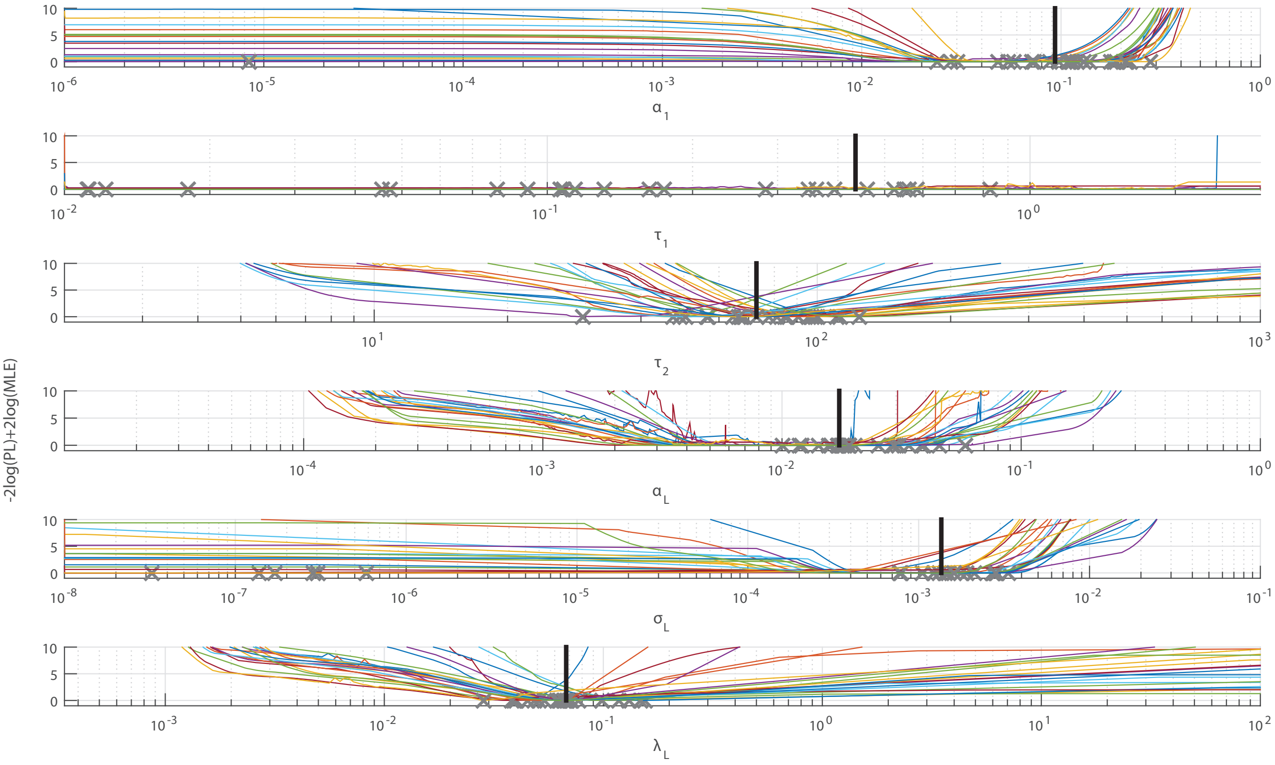

Supplementary Figure 17. PL results for the design of combinations of temporal properties $TS_{\{/2\}}$

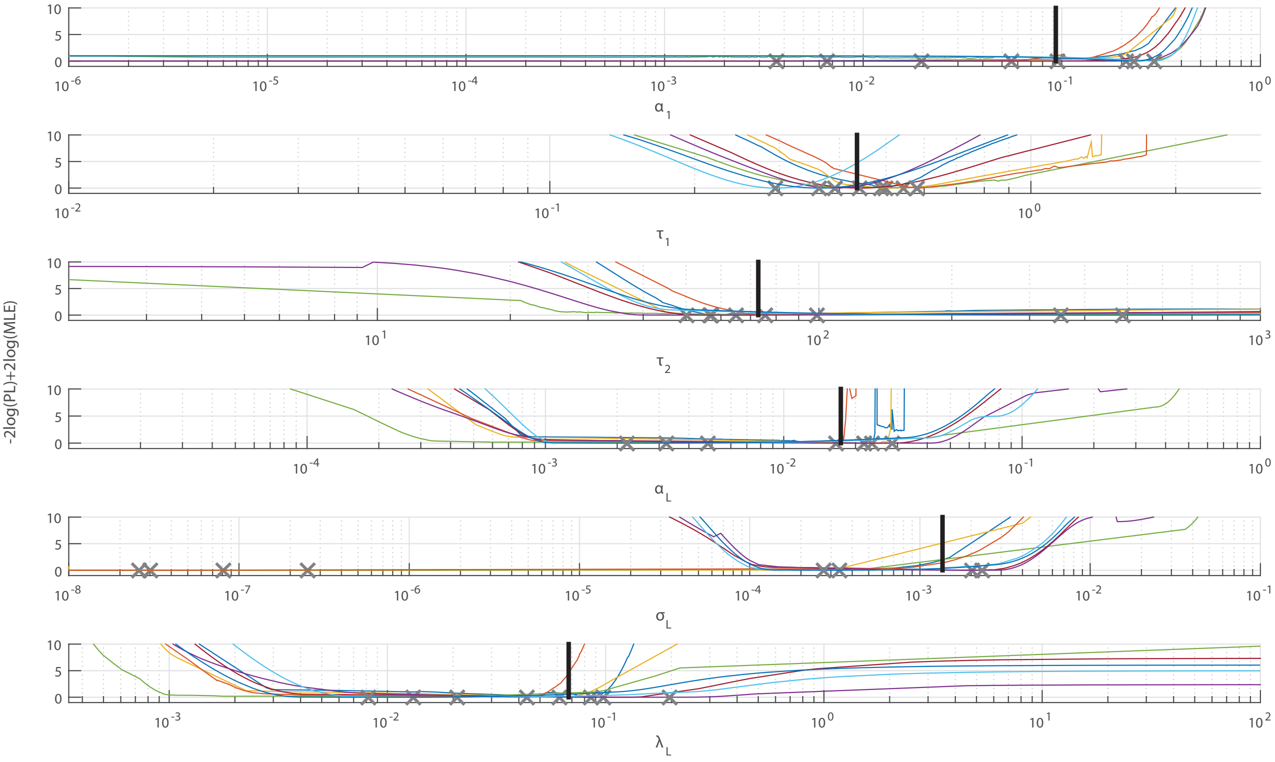

Supplementary Figure 18. PL results for the design of combinations of temporal properties $TS_{\{/3\}}$

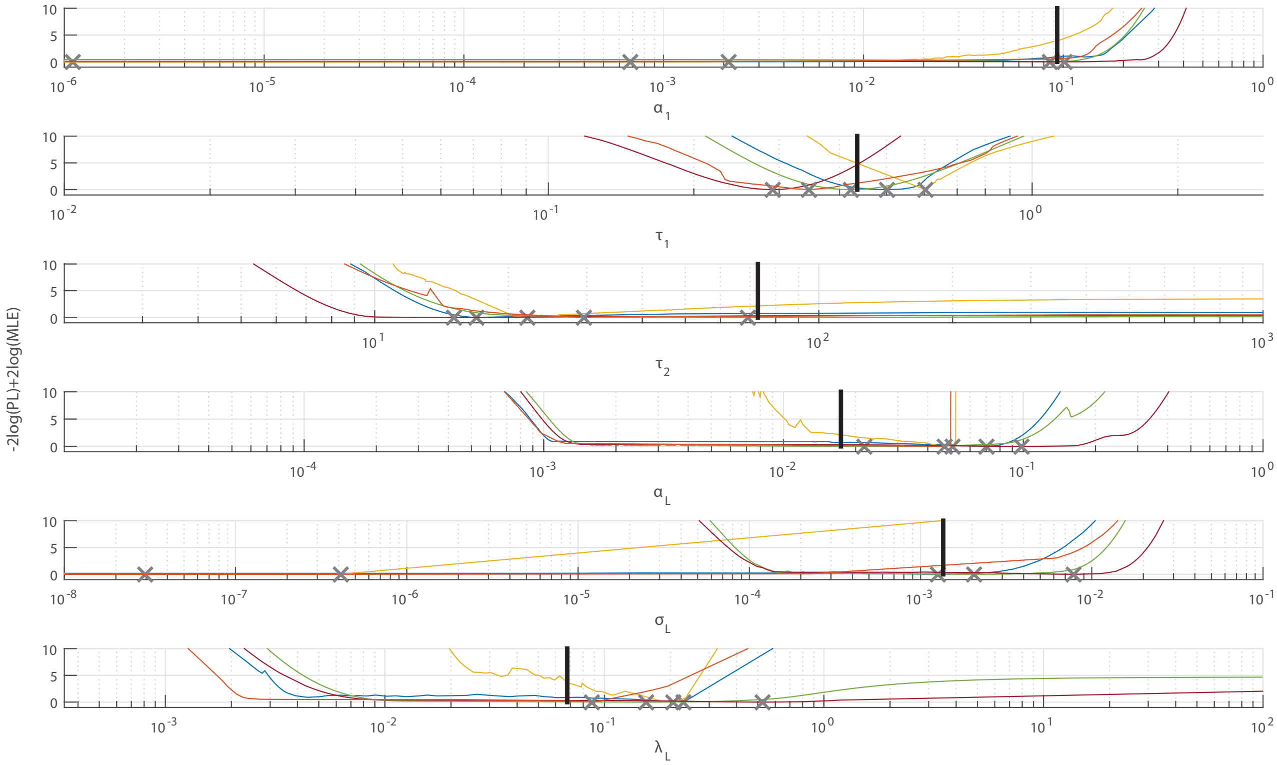

Supplementary Figure 19. PL results for the design of combinations of temporal properties $TS_{\{/4\}}$

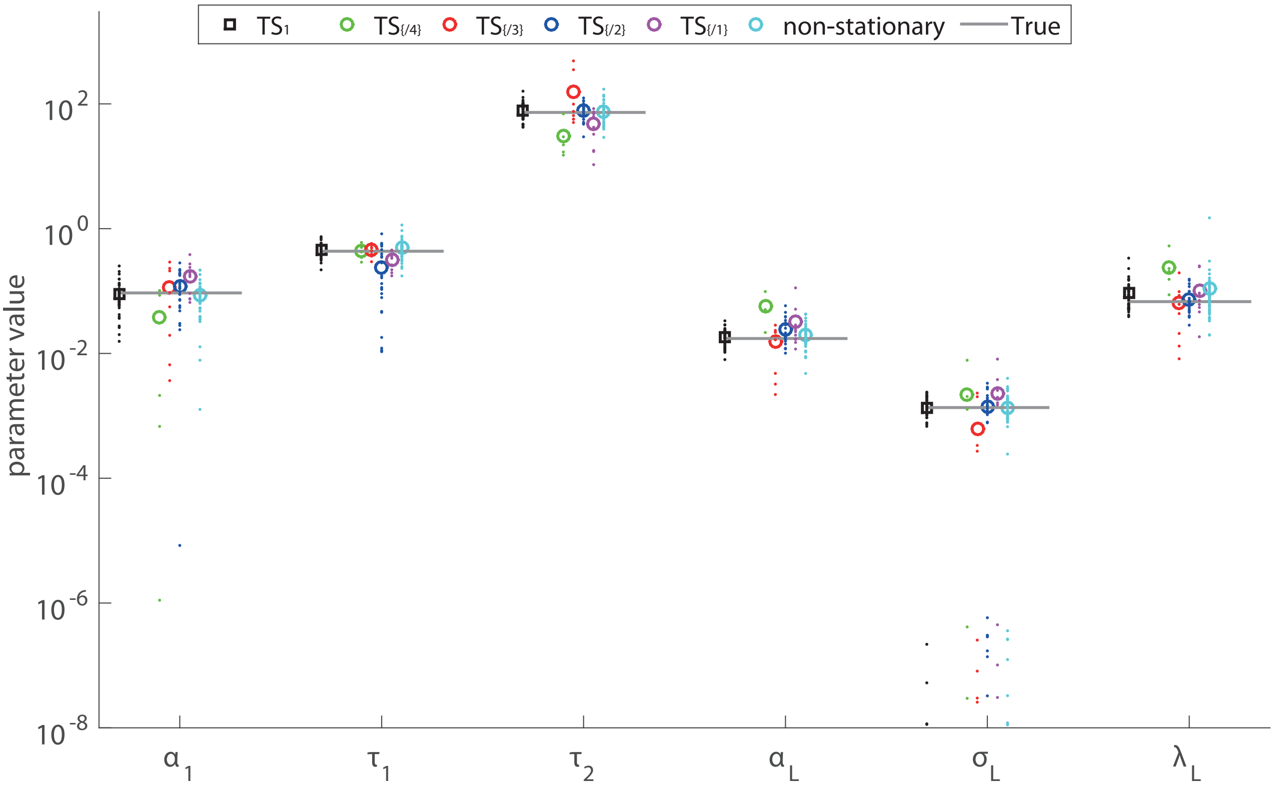

Supplementary Figure 20. Scatter plots of MLEs from different situations with subspace of the temporal properties or the non-stationary psychometric curve.

6.3 Effect of a hypothetical non-stationary psychometric curve

We use hypothetical time-varying psychometric curves for four combinations in TS_1. We plotted the curves at the start and at 10 minute.

For the system parameters at the beginning of the experiment $\boldsymbol{\theta}_0$, we use the estimate from existing dataset from subject D9450. We adapt the parameter α_L to a time-varying $\alpha_L(t) = \alpha_{L,0}(1 + 0.01)t$ to account for a non-stationary psychometric curve, where t is time in [min]. All other five parameters remain constant. We present four pairs of psychometric curves (at the 0 and 10 min of the experiment) in Fig. S21. The increase of detection thresholds (about 0.05-0.12 mA) from 0 to 10 minutes is slightly smaller but still comparable to other studies on a group level (0.1-0.2 mA) (Doll et al., 2014, 2016).

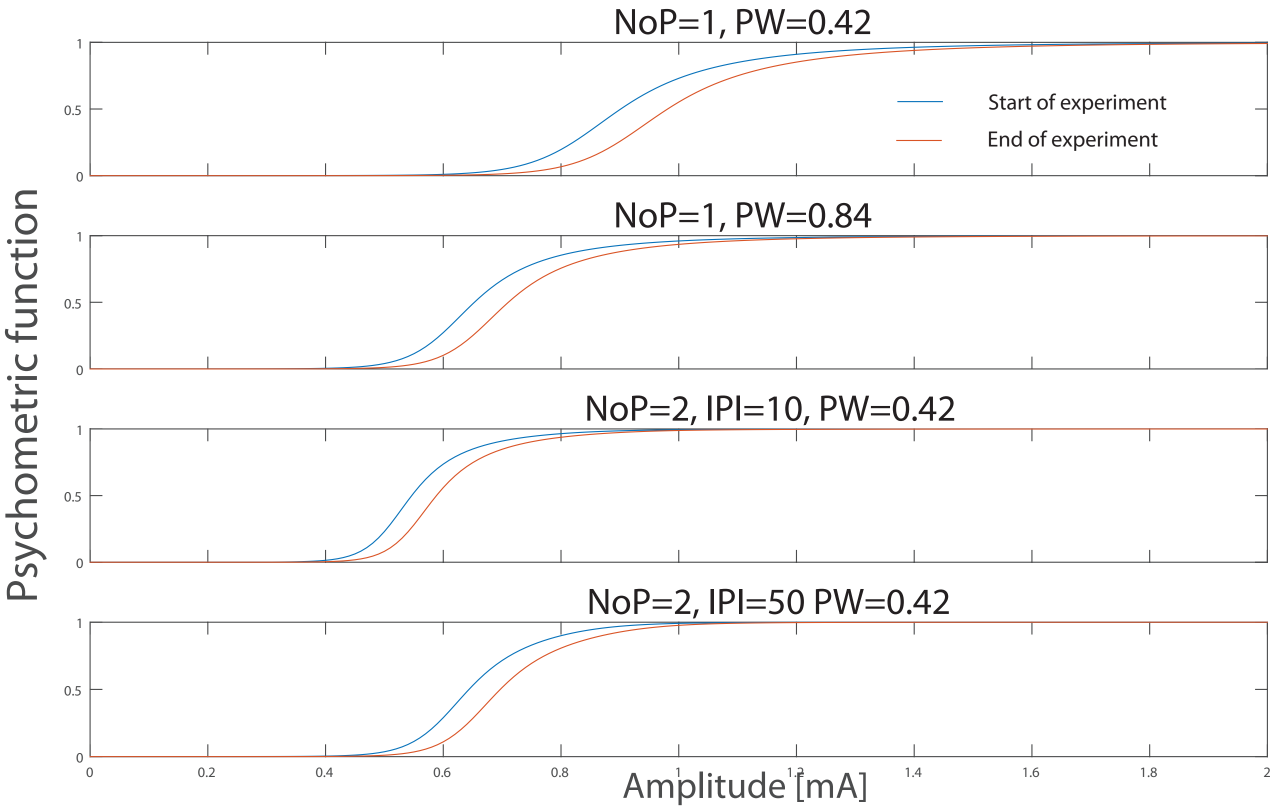

Supplementary Figure 21. Simulated non-stationary psychometric curves for four combinations of temporal properties.

Fig. S22 presents the PL results for multiple datasets with the hypothetical non-stationarity.

Fig. S20 also presents the scatter plots of MLEs for datasets with a hypothetical non-stationarity. We compare the results between stationary and non-stationary cases in Figs. S15 and S21. The widths of plots of PL are similar in general. Also, in the scatter plots in Fig. S20, the two sets of MLEs are also similarly distributed.

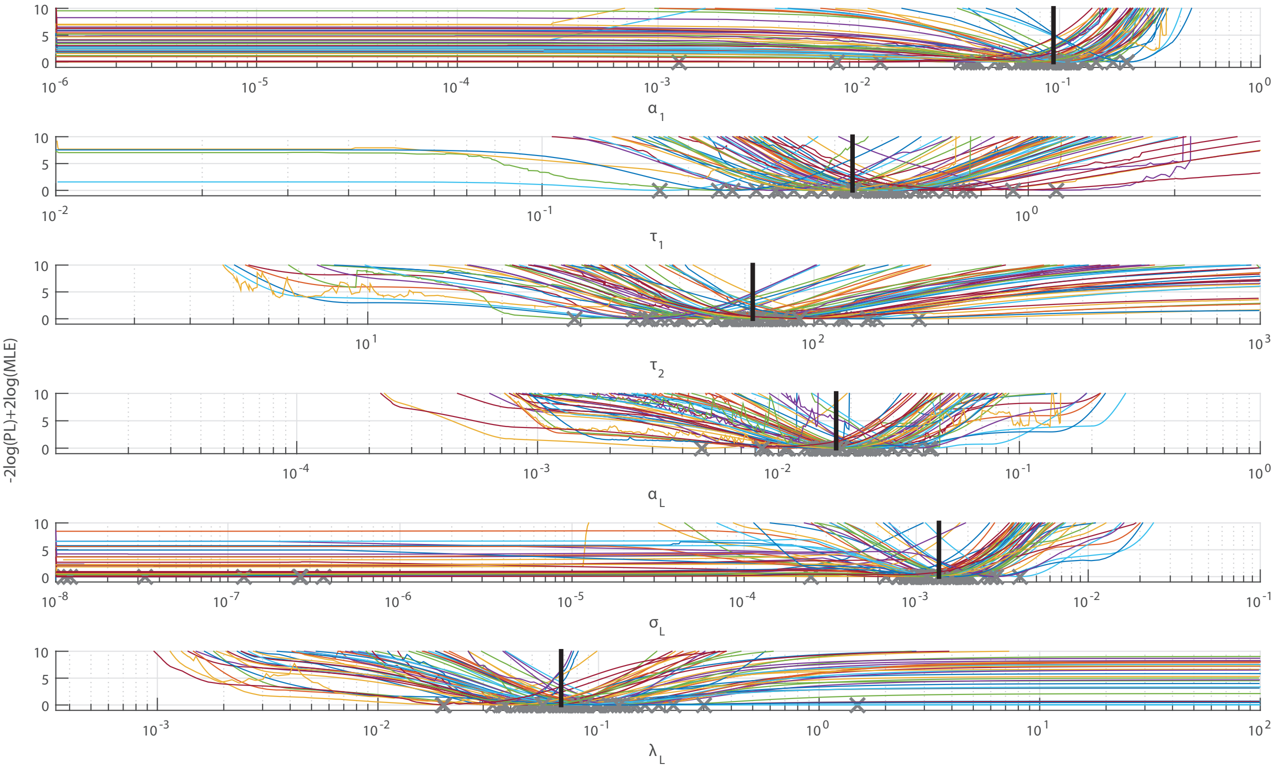

Supplementary Figure 22. With non-stationary psychometric curves, PL results for with design TS_1

REFERENCES

Doll, R.J., Buitenweg, J.R., Meijer, H.G.E., and Veltink, P.H. (2014). Tracking of nociceptive thresholds using adaptive psychophysical methods. Behavior Research Methods 46, 55–66. doi:10.3758/s13428-013-0368-4

Doll, R.J., Maten, A.C.A., Spaan, P.G., Veltink, P.H., and Buitenweg, J.R. (2016). Effect of temporal stimulus properties on the nociceptive detection probability using intra-epidermal electrical stimulation. Experimental brain research 234, 219–227. doi:10.1007/s00221-015-4451-1

Fröhlich, F., Theis, F.J., and Hasenauer, J. (2014). Uncertainty analysis for non-identifiable dynamical systems: Profile likelihoods, bootstrapping and more. In Computational Methods in Systems Biology (Springer), 61–72. doi:10.1007/978-3-319-12982-2_5

Kreutz, C., Raue, A., and Timmer, J. (2012). Likelihood based observability analysis and confidence intervals for predictions of dynamic models. BMC Systems Biology 6, 120. doi:10.1186/1752-0509-6-120

Raue, A., Schilling, M., Bachmann, J., Matteson, A., Schelke, M., Kaschek, D., et al. (2013). Lessons learned from quantitative dynamical modeling in systems biology. PloS one 8, e74335. doi:10.1371/journal.pone.0074335
