# Supplementary material for: Estimation and Identifiability of Model Parameters in Human Nociceptive Processing Using Yes-No Detection Responses to Electrocutaneous Stimulation
Source: Front Psychol. 2016 Dec 5;7:1884. doi: 10.3389/fpsyg.2016.01884 (PMC5136566; doi:10.3389/fpsyg.2016.01884)
Supplement: Supplementary file 2 [file DataSheet2.ZIP › READ_ME_Instruction_about_running_matlabscripts.pdf]

Instruction about running the Matlab scripts (Matlab version 2014b)

by Huan Yang, email: h.yang-1@utwente.nl

There are two folders of Matlab scripts, i.e."Comparison_fmincon_lsqnonlin" and "Parameter_estimation_Profile_likelihood", which were uploaded as supplementary materials 2 in the revision (also accessible from a Dutch national research data server via https://www.surfdrive.nl/en). We have added comments to the matlab (sub)routines.

The former one compares two different optimization methods, i.e. lsqnonlin and fmincon. The latter one estimates the system parameters and checks the validity of the profile likelihood approach.

1. We briefly described the former folder of matlab scripts in the revised manuscript, please see the main text around line 231 (or see https://surfdrive.surf.nl/files/index.php/s/MVxlBwkX9bMSm6u , where "run_compare.m" is the main function to run).

Note that computation of "run_compare.m"can take 20-40 minutes on a PC.

2. For the latter, we described the scripts in the supplementary material 1 around line 52 or see https://surfdrive.surf.nl/files/index.php/s/4COqhoMKXNTY471. "EST_PL_20160326.m" is the main function to perform estimation and to compute the profile likelihood for each system parameter and "compute_dist_validity.m" is the main function to check the validity of using a chi2-distirbution to determine a threshold for a confidence interval).

By running "EST_PL_20160326.m", one needs to specify one dataset containing stimuli and binary responses from the detection task. One can choose one mat file in the pop-up directory, e.g. the D9450.mat in Fig 1. After that, the multiple-starting-value optimization starts and then computation profile likelihood will follow. Note that for the dataset of D9450, the computation can take about a few hours on a PC.

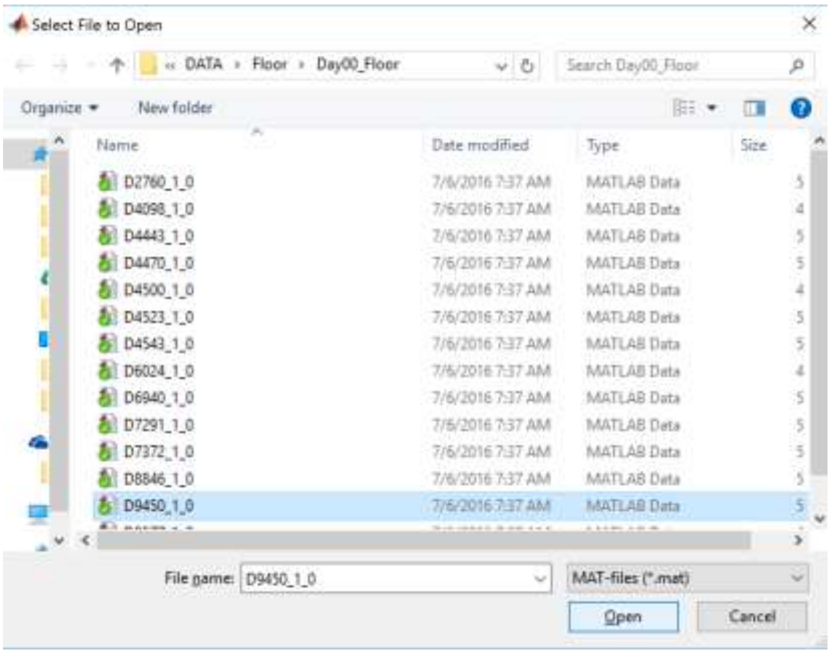

Figure 1 Choose one dataset containing the experimental stimuli and binary responses.

By running "compute_dist_validity.m", one needs to choose one experimentally obtained dataset (in the first directory). Then, in the sequentially pop-up directory, one has to choose the corresponding file containing the estimation results for the chosen experimental dataset. For example, for subject D9450, one should choose

“D9450_1_0.mat” (the same shown in Fig. 1) and “_tmp_mergedPL_D9450_1_01.mat” (shown in Fig. 2) before computation can start. The computation can take about 80 hours on a PC to generate 150 samples for a relatively smooth empirical distribution. When the computation is finished, one can run “plot_empirical_chi2.m” to visually inspect the difference between empirical distribution and the chi2 distribution.

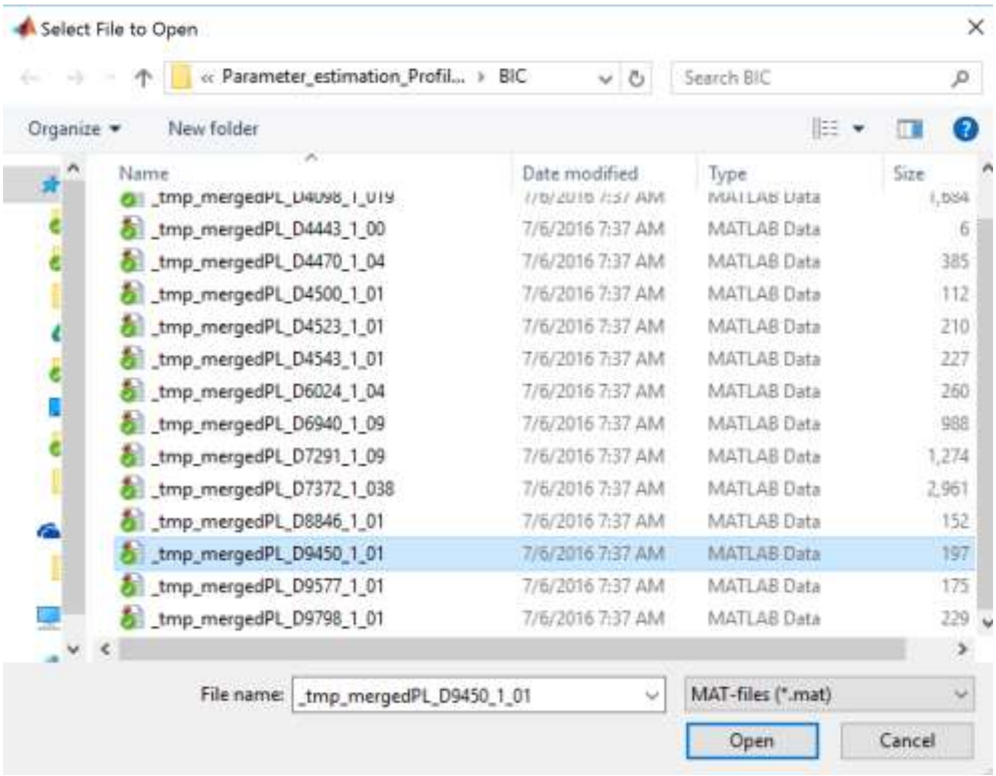

Figure 2 Choose the corresponding file containing the estimation results for the dataset D9540_1_0.
